# Proanthocyanidins from *Ginkgo biloba* extract EGb 761^®^ exert antioxidative activity *in vitro* and antiamnesic activity *in vivo*

**DOI:** 10.3389/fphar.2025.1673248

**Published:** 2025-10-27

**Authors:** Carla Sens-Albert, Markus Schmitt, Gabriele Luderer, Beatrix C. König, Silas F. Melcher, Heike Schneider, Simone Kaiser, Sabine Tremmel, Sabrina Weisenburger, Žarko Kulić, Martin D. Lehner

**Affiliations:** Preclinical R&D, Dr. Willmar Schwabe GmbH & Co. KG, Karlsruhe, Germany

**Keywords:** proanthocyanidins, Ginkgo biloba leaf extract, scopolamine, herbal medicinal products, cognitive impairment

## Abstract

**Introduction:**

Extracts from *Ginkgo biloba* leaves are widely used in the treatment of age-related cognitive decline. As regulated herbal medicinal products, these extracts are adjusted to defined contents of flavonoids and terpene lactones, which are recognized as the active constituents. Recently, proanthocyanidins (PACs) have gained increasing interest as an additional constituent group with a yet undefined role in therapeutic activity. Notably, the PAC content exhibits substantial variability across ginkgo preparations, highlighting the need for further investigation into their pharmacological relevance.

**Methods:**

In the present study, we used *in vitro* and *in vivo* assays combined with LC-(HR)-MS/MS metabolite profiling to assess the pharmacological activity, oral bioavailability, and metabolism of PACs isolated from *G. biloba* extract EGb 761^®^.

**Results:**

Ginkgo PACs concentration dependently reduced the basal cellular production of reactive oxygen species (ROS) in the rat neuronal cell line RN46A with higher potency than the ginkgo extract EGb 761^®^ itself (IC_50_ = 0.97 μg/mL vs. 3.32 μg/mL). In a T-maze model, which assessed the working memory of mice, oral pretreatment with PACs and EGb 761^®^ attenuated scopolamine-induced memory impairment with similar potency and efficacy (PAC ID_50rel_ = 30 mg/kg, I_max_ = 69%; EGb 761^®^ ID_50rel_ = 39 mg/kg, I_max_ = 68%). Gallic acid metabolites derived from PACs were detected in the plasma and urine 1 h post administration, whereas microbiota-generated metabolites of PACs were only found at later time points (6 h). The pharmacodynamic activity in the T-maze model was most prominent at 1 h post administration, indicating that the microbiota-generated metabolites did not mediate the observed pharmacological effect. Comparison of two *G. biloba* products compliant with regulatory specifications for terpene lactones and flavonoids but differing in the PAC content—high (5.0%) vs. low (0.6%)—revealed superior activity for the high-PAC formulation both in the *in vitro* ROS assay (IC_50_ = 2.54 μg/mL vs. 9.01 μg/mL) and the *in vivo* model (63% vs. 34% reversal at 50 mg/kg).

**Conclusion:**

A PAC fraction isolated from EGb 761^®^ demonstrated antioxidative activity *in vitro* and antiamnesic effects *in vivo*. These findings support the hypothesis that PACs contribute to the efficacy of EGb 761^®^.

## Introduction

*Ginkgo biloba L.,* a deciduous tree in the *Ginkgo* genus of Ginkgoaceae, is commonly considered a “living fossil” for being the oldest plant species of the gymnosperm group left after the Quaternary Glacier. *Ginkgo biloba* leaves, seeds, and exocarp have a long history of traditional use as medicine or food, with the first records dating back to Chinese documents of the Han and Song dynasties (Liu et al., 2022). Ethnopharmacologically, *G. biloba* preparations have been used for a plethora of different ailments (Liu et al., 2022). Nowadays, *G. biloba* L. (Ginkgoaceae) leaf extracts (GBEs) are among the most extensively studied and widely used herbal medicines with widespread clinical use in several different indications. These include the improvement of age-associated cognitive impairment and enhancement of the quality of life in patients with mild dementia, as outlined in the European Herbal Monograph for extract-based herbal medicinal products derived from *G. biloba* leaves (Committee On Herbal Medicinal Products, 2015), and cardiovascular and cerebrovascular disorders mentioned in the Chinese Pharmacopeia (Chinese Pharmacopoeia Commission, 2020). Much of the available clinical evidence on GBEs has been generated with the extract EGb 761^®^, which is manufactured by Dr. Willmar Schwabe GmbH & Co. KG, Germany. EGb 761^®^ is a dry extract from *G. biloba* leaves with a drug-to-extract ratio of 35–67:1 using 60% (m/m) acetone as an extraction solvent with multiple subsequent refinement steps, as reviewed by Kulic et al. (2022a). The extract is adjusted to 22%–27% ginkgo flavonoids calculated as ginkgo flavone glycosides, and 5.4%–6.6% terpene lactones consisting of 2.8%–3.4% ginkgolides A, B, and C and 2.6%–3.2% bilobalide, and it contains less than 5 ppm ginkgolic acids.

Ginkgo extract EGb 761^®^ has shown efficacy in different clinical conditions including cognitive impairment and dementia (Gauthier and Schlaefke, 2014; Tan et al., 2015; von Gunten et al., 2015; Savaskan et al., 2017), as well as tinnitus (von Boetticher, 2011) and vertigo (Hamann, 2007).

Pharmacopeial specifications for GBEs define either a concentration range or a minimum threshold for Ginkgo flavonoids and terpene lactones that are considered key contributors to the extract`s pharmacological activity (Kulic et al., 2022a). Although flavone glycosides and terpene lactones, together accounting for approximately 30% of the extract, are recognized as active constituents, they do not fully explain the overall therapeutic effects of GBEs.

Within the remaining 70% of the non-specified extract material in GBEs, proanthocyanidins (PACs) have recently gained increasing scientific interest (Zhang et al., 2023).

A PAC content of approximately 6%–7% has been reported for EGb 761^®^, with a unique composition of the PAC polymers, consisting of approximately 78% prodelphinidin, 16% procyanidin, and 3% propelargonidin as building blocks (Kulic et al., 2022b). The PAC content is in the same range as the 5.4%–6.6% terpene lactone content, indicating that PACs are a quantitatively relevant constituent of EGb 761^®^. The predominant PACs found in *G. biloba* include B-type proanthocyanidins, which are primarily composed of (epi-)gallocatechin and (epi-)catechin units linked through carbon–carbon bonds at positions 4β → 6 and 4β → 8 (Qa’dan et al., 2011). EGb 761^®^ PACs consist of polymers and dimers to approximately hexamers. The latter polymerize or aggregate in solutions to a high polymeric form over time. The polymeric proanthocyanidins form mainly delphinidin and cyanidin, trace amounts of pelargonidin, and one unknown anthocyanidin compound as the hydrolysis products (Kulic et al., 2022b). A comparative analysis of finished GBE herbal medicinal products on the German and Swiss markets using a validated method suitable for finished products containing different excipients revealed a 10- to nearly 20-fold difference in the PAC content between different products despite the adherence of all GBEs to the same regulatory specifications (Germer et al., 2024; Riesen, 2024). This difference can be attributed to the different extraction protocols for GBEs, which are all tuned toward the European Pharmacopoeia (Ph. Eur.) specifications regarding flavone glycosides and terpene lactones, rendering the remaining 70% unspecified and variable in composition (Kulic et al., 2022a).

Proanthocyanidins from different sources have been shown to exert various pharmacological effects that are relevant for the treatment of neurodegenerative diseases (Kong et al., 2017; Xu et al., 2009; Wang et al., 2012; Wang et al., 2008; Strathearn et al., 2014). PAC-enriched herbal extracts exerted neuroprotective effects against rotenone-induced neurotoxicity via the enhancement of mitochondrial function (Strathearn et al., 2014). Lotus seedpod procyanidins attenuated scopolamine-induced memory deficits in different mouse behavioral studies (Xu et al., 2009). In-depth studies on PACs from grape seed extracts reported the inhibition of amyloid beta-protein aggregation *in vitro* and the attenuation of Alzheimer disease-type cognitive deterioration in Tg2576 mice (Lian et al., 2016; Wang et al., 2012; Wang et al., 2008), along with protection against ischemia–reperfusion injury following middle cerebral arterial occlusion (Kong et al., 2017).

In line with these studies, PACs present in or isolated from *G. biloba* have also shown different pharmacological activities in several nonclinical studies. Qa’dan et al. showed that monomeric to trimeric PACs isolated from Ginkgo leaves displayed strong antioxidative activity in the 2,2-diphenyl-1-picrylhydrazyl (DPPH) assay (Qa’dan et al., 2011). Xie et al. identified catechins and procyanidins in EGb 761^®^ as constituents with high inhibitory activity against amyloid-β42 aggregation. In addition, these compounds were shown to decrease the stability of preformed amyloid beta fibers (Xie et al., 2014). In models of cerebral ischemia and reperfusion, PACs isolated from ginkgo leaves reduced the infarct volume and improved the neurological outcome upon intraperitoneal administration (Cao et al., 2016; Yao et al., 2020). Zhang et al. provided evidence of anti-inflammatory activity of ginkgo PACs in a mouse macrophage cell line by suppressing the inflammatory mediator production induced by LPS (Zhang et al., 2023). Recently, ginkgo PACs were shown to improve bioenergetics and stimulate neurite outgrowth in human neuroblastoma cells *in vitro* (Lejri et al., 2025).

Despite the increasing evidence of pharmacological activity of ginkgo PACs, data on the *in vivo* activity upon oral dosing of these usually oligo- or polymeric compounds are limited, and the contribution of PACs to the activity of GBEs remains elusive.

The recent findings that PACs are a relevant constituent of EGb 761^®^, that major differences in the PAC content exist between registered GBE medicines, and that there is increasing evidence of a potential pharmacological activity of PACs prompted us to experimentally assess whether these constituents play a role in the pharmacological activity of EGb 761^®^.

In the present study, we employed a combination of *in vitro* and *in vivo* pharmacological assays and *ex vivo* LC-(HR)MS/MS metabolomics to study the activity and metabolite profiles after the administration of EGb 761^®^ and an isolated PAC fraction. In addition, we conducted head-to-head comparison studies of two commercial products to assess the relevance of differing PAC contents for ginkgo extract activity.

Our data provide novel insights into the pharmacological activity and metabolic fate of ginkgo PACs and their contribution to the activity profile of EGb 761^®^.

## Materials and methods

### Ginkgo extract, isolated fractions, and finished products

*G. biloba* leaf extract EGb 761^®^ was obtained from a production batch (WS1133/EXCh.053) with the pharmaceutical specifications as indicated above. A voucher specimen was deposited at Dr. Willmar Schwabe GmbH & Co. KG. The isolation protocol and phytochemical characterization of the ginkgo PAC fraction used in the present study have been described by Kulic et al. (2022b). From the same EGb 761^®^ batch, terpene lactones and flavonoid fractions were isolated by the same protocol as for the PAC fraction, albeit different fractions were collected. Briefly, 1 kg of the extract was dissolved in 2 L deionized water at 60 °C. After cooling to room temperature, the solution was filtered through a 10 µm–16 µm glass filter. The insoluble residue was suspended in approximately 100 mL deionized water, sonicated for 5 min, and filtered again. The remaining insoluble residue consisted mainly of ginkgolides. An amount of 1.54 g of this fraction was reconstituted with 0.46 g bilobalide to yield the terpene lactone fraction. Analytical characterization was conducted according to the quantification method of the European Pharmacopoeia (Ph. Eur.) and revealed contents of 23.0% bilobalide, 8.5% ginkgolide A, 7.6% ginkgolide B, and 7.6% ginkgolide C. The aforementioned fractionation protocol was continued with the water-soluble ginkgo extract by fractionating with a stepwise gradient, as described by Kulic et al. (2022b), to yield the PAC fraction and multiple flavonoid fractions. The flavonoid fractions were identified by thin-layer chromatography for ginkgo flavonoids, as described in the Ph. Eur. Fractions containing orange and green spots corresponding to quercetin- and kaempferol-based flavonol glycosides, respectively, were collected and reconstituted to yield a ginkgo flavone glycoside-enriched fraction. The analytical characterization of this fraction based on the Ph. Eur. method for ginkgo extracts showed a ginkgo flavone glycoside content of 48.79%. Terpene lactones and PACs were not detectable in this fraction termed the “flavone fraction.”

For comparison of GBEs with different PAC contents, 4–6 batches of commercially available herbal medicinal products were purchased from German pharmacies in 2019–2020 and analyzed for the PAC content according to the study by Germer et al. (2024). Four different batches each of Tebonin^®^, konzent^®^ 240 mg (Dr. Willmar Schwabe GmbH & Co. KG), and Kaveri^®^ 120 mg (KSK-Pharma) were used for the *in vitro* reactive oxygen species (ROS) assay, and three were used for assessing *in vivo* antiamnesic activity in the T-maze model, respectively. The Tebonin^®^ batches used in our study (0070320, 0080320, 2190819, 2280917, 2811218, and 0220121) showed a mean PAC content of 5.0%, whereas the Kaveri^®^ batches (001106, 173084, 181120, and 192057) had a PAC content of 0.6%. In addition to the difference in the PAC quantity, the delphinidin-to-cyanidin ratio was different between the test products (Tebonin^®^: D:C 6.94 ± 0.37:1; Kaveri^®^: 3.35 ± 0.16:1). All pharmacological studies involving commercially available products were conducted before June 2021 within the shelf-life time of the products. Exemplary HPLC chromatographs are shown in Supplementary Figure S1, and a summary of delphinidin-to-cyanidin ratios is provided in Supplementary Figure S2.

For pharmacological studies, finished products were ground to powder with a mortar and pestle and suspended in the vehicle (cell culture media for *in vitro* experiments and 0.2% agar for *in vivo* experiments).

## Animals

All studies were performed in accordance with regulations for animal welfare, and the experimental protocols were approved by the responsible regional council (Regierungspraesidium Karlsruhe, Land Baden-Wuerttemberg, Karlsruhe, Germany) under the following reference numbers: A-43/17, A-36/17, and G-108/20. Male NMRI mice (Crl:NMRI(Han)) at 4–5 weeks of age were obtained from Charles River (Sulzfeld, Germany). Male SD rats (RjHan:SD) at 5 weeks of age were obtained from Janvier Labs (Le Genest-Saint-Isle, France). Animals were housed in groups of 2–3 with free access to food and water and were supplied with environmental enrichment. Prior to the administration of test substances, the animals were fed a flavone-free diet (AIN 93G, Sniff) for 1 week for behavioral experiments and for 3 weeks for kinetic studies. Animals were maintained under artificial lighting (12 h) between 6:00 a.m. and 6:00 p.m. at a controlled ambient temperature of 22 °C and a relative humidity of 55%.

### T-maze spontaneous alternation

**FIGURE 1:**
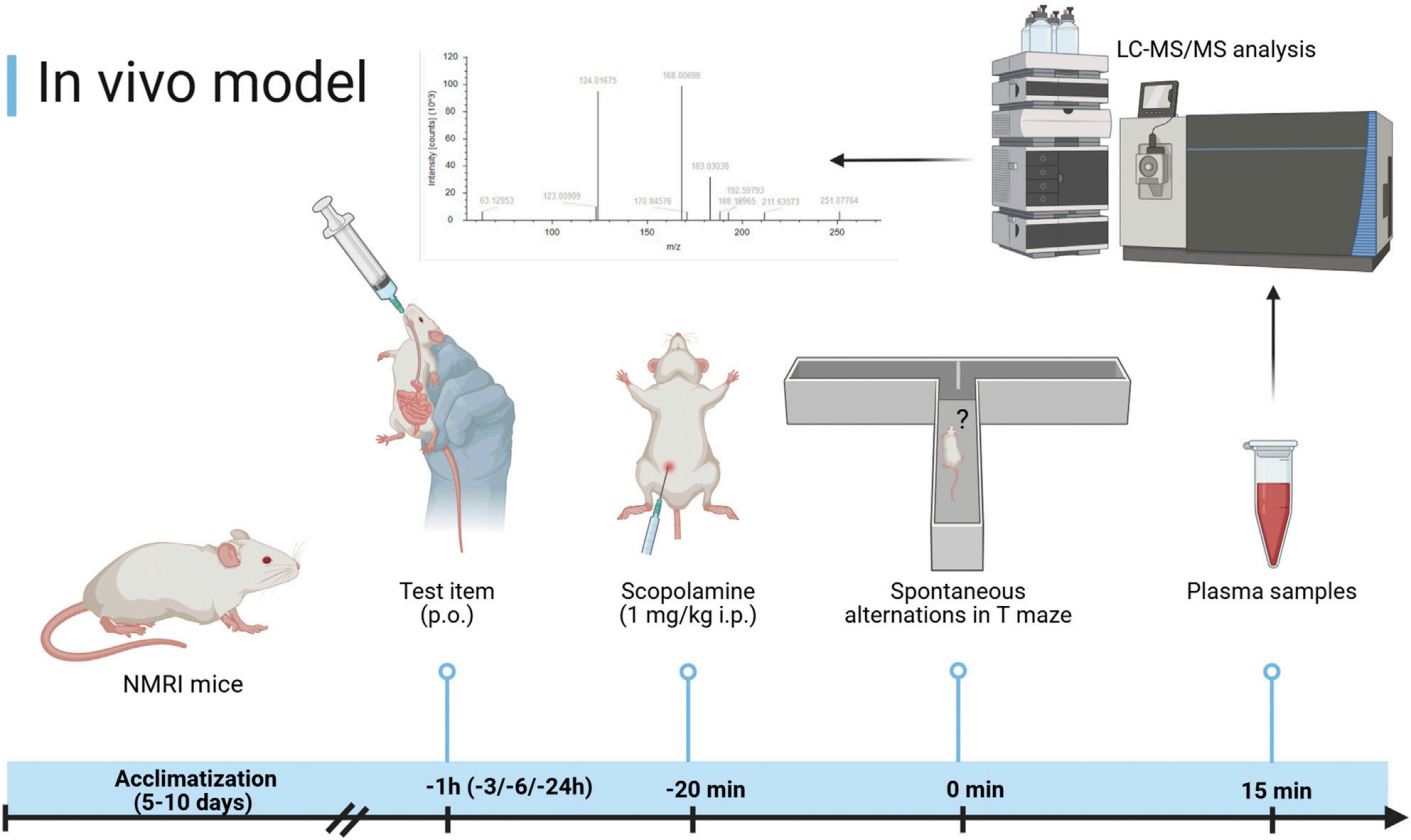
Experimental setup of the T-maze spontaneous alternation model. Created in BioRender. Lehner (2025). https://BioRender.com/swfln69.

One hour before testing spontaneous alternation in the T-maze, mice received perorally the vehicle (0.2% agar, 6494.1, Carl Roth), donepezil (0.3 mg/kg, D6821, Sigma-Aldrich), EGb 761^®^ (10 mg/kg–300 mg/kg), isolated fractions of PACs (3 mg/kg–300 mg/kg), terpene lactones (1 mg/kg–100 mg/kg), flavones (10 mg/kg–300 mg/kg), or two finished commercial products with different ginkgo extracts (50 mg/kg). To impair short-term memory, mice were injected intraperitoneally (i.p.) with 1 mg/kg scopolamine (S1875, Sigma-Aldrich) or 0.9% NaCl (PZN01399062, Fresenius) 20 min prior to the experimental session. Mice were observed for 20 arm entry trials with documentation of the explorative behavior. Switching from the right to the left arm of the T-maze or *vice versa* was calculated as correct alternations (%). The method is based on the study by Deacon (2006). At 15 min after the start of the T-maze observation and once the 20 arm entry trials were finalized, animals were euthanized with CO_2_, and blood was collected by cardiac puncture for EDTA-plasma (Figure 1).

For studying the relation of the plasma concentration of different constituents and metabolites with antiamnesic activity, we conducted a pharmacokinetic–pharmacodynamic (PK–PD) relation study. To this aim, we utilized the same protocol as for the abovementioned scopolamine-induced amnesia model but used different pretreatment times for the vehicle, donepezil (0.3 mg/kg), EGb 761^®^ (100 mg/kg), and PACs (100 mg/kg) of 1 h, 3 h, 6 h, or 24 h. Six mice per test item and pretreatment time were used for the analysis of antiamnesic activity in the T-maze model. After completion of the T-maze test, 35 min after the administration of scopolamine, the animals were sacrificed with CO_2_, and blood was drawn by cardiac puncture for the preparation of EDTA-plasma.

### Rat PK study

Animals (n = 3 per time point) received oral administration of vehicle (agar 0.2%, 10 mL/kg) or test items (EGb 761^®^ at 300 mg/kg; PAC fraction isolated from EGb 761^®^ at 300 mg/kg) via gavage at t = 0 h. Animals to be sacrificed at 6 h (only test item-treated animals) were placed in metabolic cages (650-0100, Nalgene) for the collection of urine. Urine was stabilized with a final concentration of 4 mM NaN_3_ and stored at 4 °C. At 1 h and 6 h after the administration of the vehicle (1 h only) or test items (1 and 6 h), animals from the respective treatment groups were euthanized by CO_2_ inhalation. Blood was obtained by cardiac puncture and drawn in lithium-heparin-containing vials for the preparation of plasma by centrifugation (4,000 rpm, 4 °C, 10 min). Plasma was stored at −80 °C until bioanalytical analysis. Samples of the mixed intestinal content were obtained by scraping the small intestine, cecum, and large intestine into a 5-mL tube. Tubes were covered with gaseous N_2_ and stored at −80 °C.

### Source and culturing of RN46A cells

The immortalized rat serotonergic neuronal cell line (RN46A) was purchased from ECACC (General Collection No. 12061302). Cells were cultured on Poly L-Lysin-coated (P4832, Sigma-Aldrich) culture plates in DMEM/F12 (1:1) (D8062, Sigma-Aldrich) containing 10% FCS (F7524, Sigma-Aldrich) + 2 mM Glutamine (592020C, Sigma-Aldrich) + 250 μg/mL G418 (ant-gn, InvivoGen) and 1% antibiotic–antimycotic solution (A5955, Sigma-Aldrich). Cells were regularly negative-tested for *mycoplasma* contamination using a MycoStrip™ *Mycoplasma* Detection Kit (MycoStrip™ 100, InvivoGen).

### Reactive oxygen species (ROS) assay

RN46A cells were seeded at 25.000 cells per well in Corning^®^ 96-well Flat Clear-Bottom Black Polystyrene TC-treated Microplates (3603, Corning) and cultured for 24 h. The medium was removed, and cells were loaded with 25 µM DCFDA/H2DCFDA (ab113851, abcam) for 45 min followed by treatment with either a serial dilution of EGb 761^®^, its purified fractions of PACs, terpene lactones, or flavones (0.03 μg/mL, 0.1 μg/mL, 0.3 μg/mL, 1 μg/mL, 3 μg/mL, 10 μg/mL, 30 μg/mL, and 100 μg/mL), or a similar dilution series of the finished products with a high PAC content (Tebonin^®^), a low PAC content (Kaveri^®^), or the corresponding excipients for Tebonin^®^. DMSO was used as the solvent and added accordingly at 0.1% as the control treatment. Dilutions for treatment were prepared in the absence of Phenol red in Dulbecco’s Modified Eagle’s Medium/Nutrient Mixture F-12 Ham (D-6434, Sigma-Aldrich). 2′,7′–Dichlorofluorescein (DCF) production was measured 1 h after adding the treatment at 535 nm using a SpectraMax M Multimode Microplate Reader (Molecular Devices).

### Evaluation of the test item effects on cell proliferation, cell cycle, and interference with DCF fluorescence

Effects of the treatments on cell cycle progression and proliferation in RN46A cells were conducted for the unfractioned ginkgo extract EGb 761^®^ and isolated PAC fraction. The detailed protocols are included in the Supplementary Material.

To rule out the potential interference (“quenching”) of test items with DCF fluorescence, incubations were conducted by adding different concentrations of test items to a DCF solution, followed by the determination of fluorescence. The detailed protocol is included in the Supplementary Material.

### Acetylcholine esterase assay

To evaluate the direct inhibitory effects on acetylcholine esterase (AChE) activity, we adapted a protocol from the study by Ellman et al. (1961). The detailed protocol is included in the Supplementary Material.

### Quantification of acetylcholine in plasma

The Amplex^®^ Red Acetylcholine/Acetylcholinesterase Kit (A12217, Invitrogen) was utilized to assess acetylcholine (ACh) concentration in plasma samples from animals used in the T-maze model. The detailed protocol is included in the Supplementary Material.

### Metabolite profiling

#### Sample preparation

Protein precipitation for plasma: briefly, 300 µL of plasma was diluted with 1,000 µL 1% formic acid (FA) (84865.180, VWR) in acetonitrile (ACN) (hypergrade for LC-MS LiChrosolv, 1.00029.1000, VWR) on ice, vortexed, sonicated, and centrifuged. The supernatant was evaporated to dryness and resuspended in 150 µL 1% FA in H_2_O/ACN (9/1, v/v) for analysis.

Rat intestinal content was first freeze-dried and finely crushed. An amount of 100 mg was extracted twice with 1 mL ACN (+2 mg/mL DTT (6908.1, Roth) as the antioxidant)/5 mM Tris-HCl (1.08382.0500, Merck) buffer (1/1, v/v) adjusted to pH 7.3 with 1H HCl on ice. Then, the organic phase was evaporated, and metabolites were extracted from the aqueous phase three times with ethyl acetate (LiChrosolv^®^ for HPLC, 1.00868.1000, VWR) (pH adjusted to 3 with FA) on ice. Ethyl acetate extracts were evaporated to dryness and then resuspended in 300 µL 1% FA in H_2_O/ACN (9/1, v/v) for untargeted LC-HRMS analysis. Internal standards (listed in Supplementary Table S1) were spiked to both the sample types to ensure the performance of the measurements and monitor matrix effect.

### Untargeted LC-HRMS analysis

Untargeted metabolite profiling was performed using a Vanquish UHPLC coupled with an Orbitrap Fusion Tribrid mass spectrometer (Thermo Fisher Scientific, Waltham, MA, United States of America) fitted with a HESI source. Chromatographic separation was performed on Waters Acquity HSS T3 1.8 µm Column (2.1 mm × 150 mm) (Waters Technologies, United States of America). The injection volume was 5 µL. The flow rate was set at 0.45 mL/min, and the column temperature was 40 °C. The mobile phases consisted of 0.1% FA in water with 2.5% ACN (phase A) and 0.1% FA in ACN with 2.5% water (phase B). The gradient was as follows: 0% B from 0 to 2 min, 0%–10% B from 2 to 10 min, 10%–80% B from 10 to 25 min, 80%–100% B from 25 to 26 min, 100% B from 26 to 31 min, 100%–0% B from 31 to 32 min, and 3 min equilibration at 0% B.

The positive and negative ionization modes were used in two runs. Source parameters were set as follows: spray voltage at + 3,500 V and −2,500 V, sheath gas at 50 (arbitrary units), auxiliary gas at 10 (arbitrary units), sweep gas at 1 (arbitrary units), ion transfer tube temperature at 325 °C, and vaporizer temperature at 350 °C. Ion transfer parameters were set as follows: mass range at “normal” and S lens RF level at 60%. Scan parameters applied were as follows: acquisition time from 0 to 25 min, micro scan at “1,” data type at “profile,” and automatic gain control target standard. For acquisition in the MS mode only, the data were acquired using the Orbitrap analyzer at a resolution of 120,000; the maximum injection time was set to auto, and the scan range was set from m/z 100 to m/z 1,000. The 10 most abundant ions of the full-scan spectrum were sequentially fragmented. To exclude background signals, first, a solvent blank sample was analyzed prior to each acquisition batch to (manually) generate an exclusion list from the detected background signals present at rather high intensities (100 most abundant masses) with a mass tolerance of 5 ppm. Target ions (produced from relevant metabolites) already selected for MS2 were dynamically excluded after two selections for 4 s (“dynamic exclusion” filter) with a mass tolerance of 2.5 ppm. Similarly, the “apex detection” filter was used for selecting an m/z signal at its highest intensity. “Monoisotopic precursor selection” (MIPS) filter was used to avoid the selection of 13C isotopes. Selected ions were fragmented with CID (normalized collision energy, 30%) and analyzed in the linear ion trap (isolation width was 0.7) and with HCD (higher-energy collisional dissociation, 30%, 50%, and 70%) in the Orbitrap.

### Data processing with compound discoverer

Compound discoverer 3.1 (CD, Thermo Fisher Scientific, Waltham, MA, United States of America) was used for untargeted and novel feature detection. Raw files were processed with CD in the following steps: first, spectra were selected from raw data followed by retention time (RT) alignment with RT tolerance of 2 min and 5 ppm mass precision. Then, unknown compounds were detected and grouped with RT tolerance of 0.5 min and 5 ppm mass deviation. Missing values were filled. The composition was predicted based on the exact mass. The compounds were annotated with different types of databases. mzCloud was used to annotate compounds on the MS/MS level with a mass tolerance of 10 ppm. ChemSpider was used to annotate features based on the exact mass with a mass tolerance of 20 ppm.

Relevant features were identified by performing differential analysis between the vehicle and PAC treatment groups in Compound Discoverer software. Only upregulated features of the treatment group in the volcano plot were considered for further analysis: Log_2_ fold change of >2 and -Log_10_ p-value of >2. Compound annotations of these features were checked for plausibility, and, if necessary, structural elucidation was manually continued.

The confidence level of annotation was created based on the study by Schymanski et al. (2014).

### Targeted analysis with LC-MS/MS

Targeted LC-MS/MS was used to quantify known and newly identified PAC metabolites (MRM transitions based on MS2 spectra of the untargeted analysis), as well as main ginkgo terpene lactones and flavonol aglycones in one single run in mouse plasma obtained from the pharmacological experiments (Supplementary Table S1).

The LC instrument setup consisted of an Agilent 1290 Infinity II HPLC and an Agilent 6470A Triple Quadrupole MS/MS. The injection volume was 10 µL. Chromatographic separation was performed with a Waters Acquity UPLC high-strength silica (HSS) T3 1.8 µm (2.1 mm × 150 mm) column (Waters, Wilmslow, United Kingdom). The gradient conditions of the mobile phase were as follows: 0 min–2 min 0% B; 2 min–10 min 0%–10% B; 10 min–25 min 10%–80% B; 25 min–26 min 80%–100% B, 26 min–31 min 100% B, 31 min–32 100–0 %B, and 32 min–35 min 0% B. The source conditions were as follows: gas temperature of 210 °C, flow rate of 4 L min^−1^, capillary voltage of 2,000 V (−), nozzle voltage of 0 V, sheath gas temperature of 400 °C, and sheath gas flow of 12 L min^−1^. Target compounds were analyzed in the ESI-/ESI + ionization mode with multiple reaction monitoring (MRM) using tandem mass spectroscopy (MS/MS) transitions (see Supplementary Table S1). Data processing was carried out with MassHunter Quantitative Analysis 10.1 software.

### Quantification of plasma levels of terpene lactones and flavonol aglycones

Plasma levels of terpene lactones (ginkgolide A, ginkgolide B, and bilobalide) and main ginkgo flavonol aglycones (quercetin, kaempferol, and isorhamnetin) were quantified in samples from animals used in the T-maze model. The detailed protocols for the analysis methods are included in the Supplementary Material.

### Data analysis and presentation

Data were analyzed using GraphPad Prism 10.1.2 (GraphPad Software, La Jolla, CA). Experimental groups were compared using one-way analysis of variance (ANOVA) or two-way ANOVA, followed by Dunnett’s post-test. Vehicle and each active treatment group were compared to the vehicle–scopolamine group (that received vehicle p.o., followed by i.p. injection of scopolamine). For calculation of dose–response curves, reversal of the scopolamine-induced amnesia was calculated. To this aim, the percent correct alternation values from the T-maze studies were normalized to the control values set as 100% and the scopolamine group values set as 0% inhibition. For ROS data, baseline ROS levels without treatment were set as 0% inhibition. Log-transformed dose or concentration values vs. the mean response values were used for the calculation of dose–responses with nonlinear regression analysis (variable slope, four parameters). For T-maze data, the bottom value of the curves was constrained at 0. E_max_, and relative ID_50_ values for the T-maze *in vivo* studies were taken directly from the calculated dose–response curve data provided by Graph Pad Prism. Absolute IC_50_ values (ROS *in vitro* studies) were calculated by interpolation (calculation of the concentration corresponding to 50% inhibition), followed by anti-log transformation. The relevant p-values <0.05 are presented in the figures.

## Results

### PACs and ginkgo extracts exert antioxidative activity *in vitro*

**FIGURE 2:**
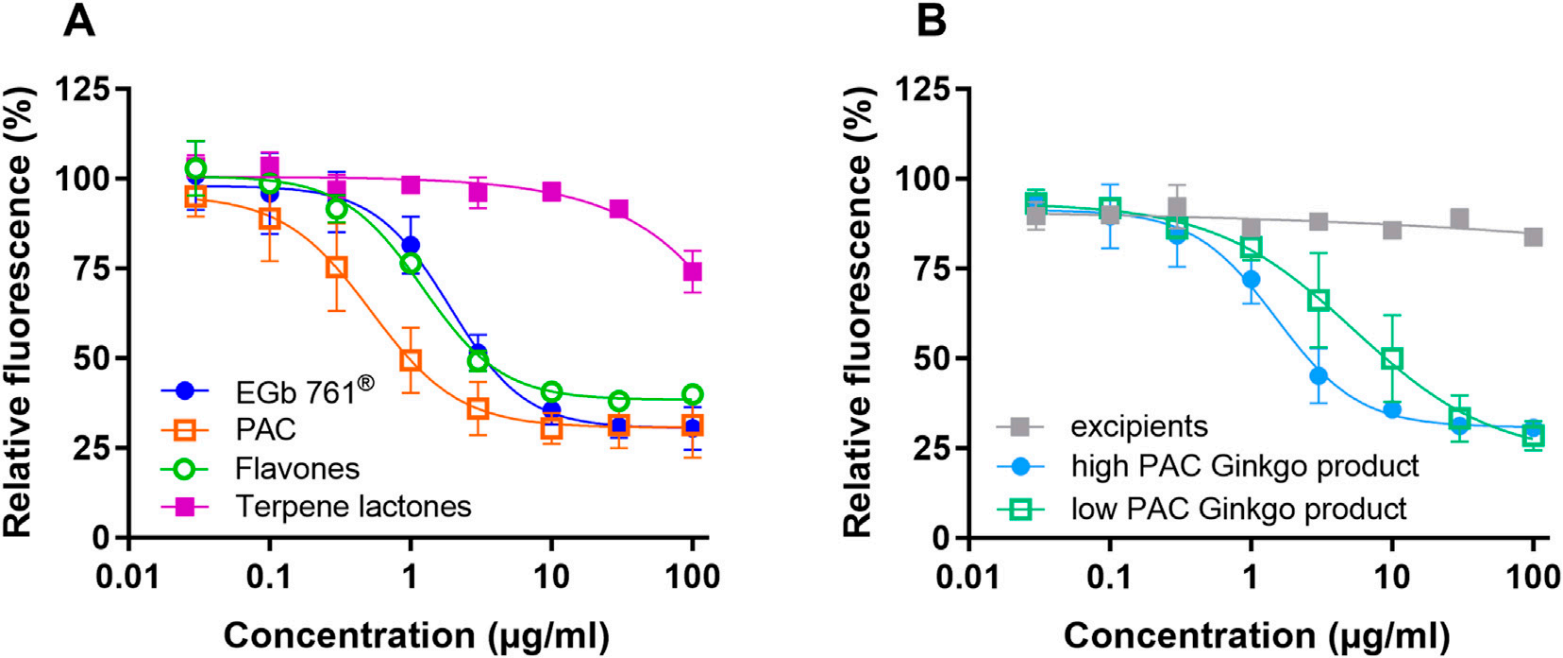
Inhibition of intracellular basal ROS production in rat neuronal RN46A cells. **(A)** Concentration–response curves of EGb 761^®^ (n = 8) and isolated fractions of PAC (n = 5), flavone glycosides (n = 2), and terpene lactones (n = 3). **(B)** Concentration–response curves of two finished ginkgo products (n = 4 different product batches, each) and excipients without the extract (n = 2). Data are shown as the means ± SD.

Antioxidative activity has been reported for GBEs and some of the compound classes contained in GBEs such as flavonoids and PACs. To assess the potential contribution of PACs as well as the canonical active constituents—terpene lactones and flavones—to the antioxidative activity of EGb 761^®^, rat serotonergic neuronal cells (RN46A) were loaded with DCFDH/H_2_DCFDA and subsequently treated for 1 h with EGb 761^®^ or the isolated PAC, terpene lactone, and flavone fractions. Except for the terpene lactone fraction, the test items concentration-dependently reduced the basal ROS production assessed by DCF fluorescence (Figure 2A). The isolated PAC fraction (IC_50_ = 0.97 μg/mL) showed an approximately 3-fold higher potency than the whole extract EGb 761^®^ (IC_50_ = 3.32 μg/mL) and the flavone fraction (IC_50_ = 3.19 μg/mL). The low IC_50_ value of the isolated PAC fraction suggested that PACs could contribute to the antioxidative activity of EGb 761^®^. The potential interference with DCF fluorescence or cytotoxicity was excluded in separate experimental approaches (Supplementary Figures S3, S4, S5).

To assess the relevance of PACs for the antioxidative activity of GBEs, we tested ground tablets of two commercial herbal medicinal products (four different batches each) containing Ph. Eur. quality GBEs (and thus, similar amounts of terpene lactones and flavone glycosides) with different PAC contents. We also tested tablet excipients available for one of the products for potential interference and observed no relevant effect on fluorescence in the assay. This observation indicated that the use of finished products was feasible for the unbiased detection of the active constituents. Both products showed antioxidative effects, as would be expected from their similar content of flavone glycosides. However, the high-PAC GBE product showed a more potent inhibition of ROS production than the low-PAC product (IC_50_ = 2.54 μg/mL vs. 9.01 μg/mL, Figure 2B).

### Oral administration of EGb 761^®^ and fractions of terpene lactones and PACs, but not flavones, attenuated scopolamine-induced cognitive impairment

**FIGURE 3:**
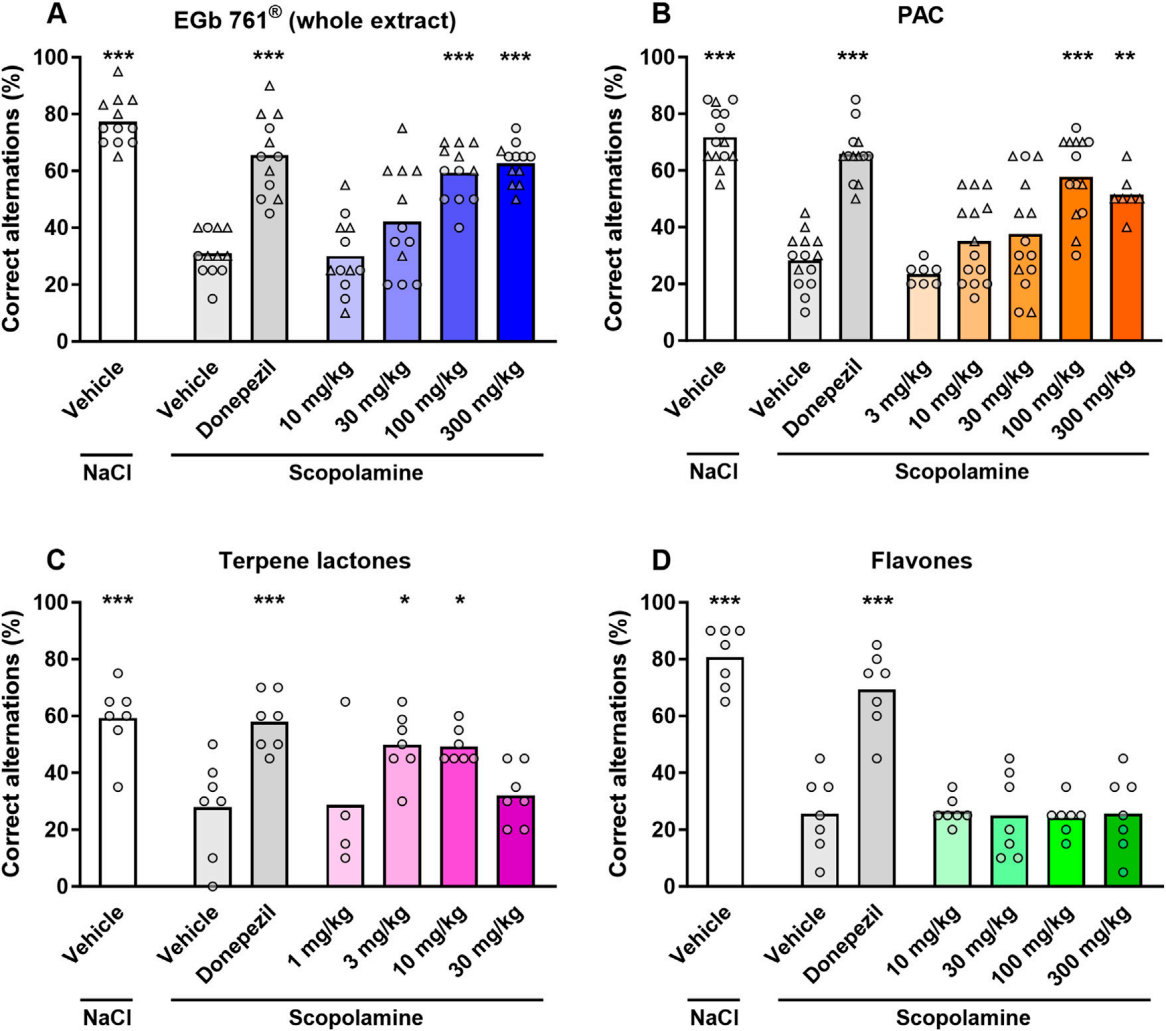
Reversal of scopolamine-induced working memory impairment in the T-maze test. Percentage of correct spontaneous alternations (n = 20 arm entries) after impairment of working memory by i.p. injection of scopolamine (1 mg/kg) was reversed by oral pretreatment effect of EGb 761^®^ (n = 12) **(A)**, the PAC fraction (n = 7–14) **(B)**, and the terpene lactone fraction (n = 4–7) **(C)**, but not by the flavone fraction (n = 7) **(D)**. Donepezil (0.3 mg/kg p.o.) was used as the positive treatment control. Data are presented as the means and individual values; *p < 0.05 and ***p < 0.001 vs. vehicle–scopolamine group based on one-way ANOVA with Dunnett’s test.

**FIGURE 4:**
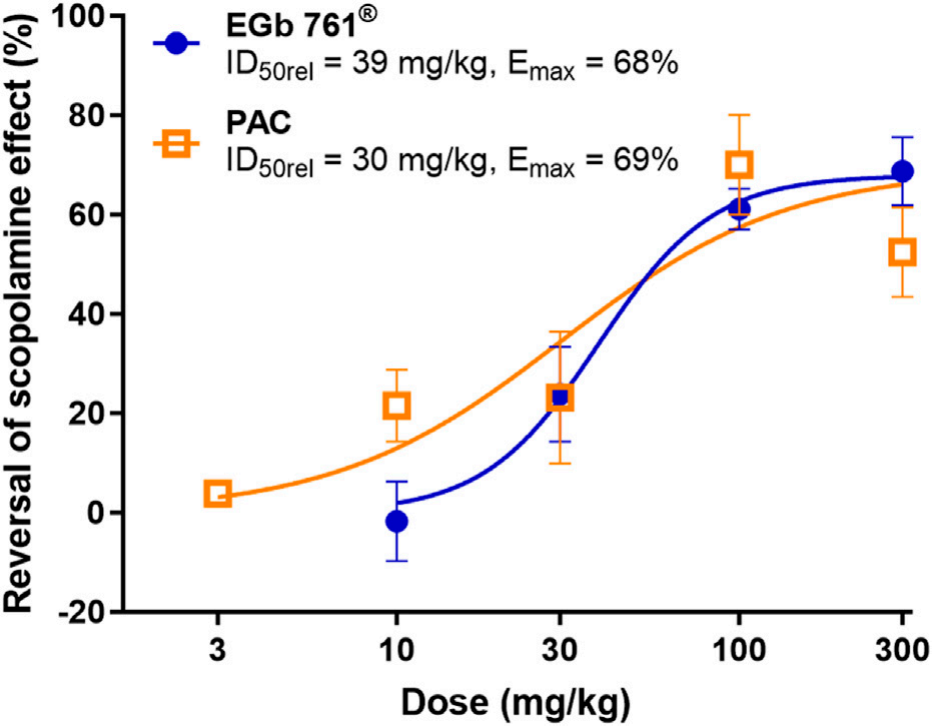
Concentration-dependent reversal of scopolamine effect. Dose–response curves calculated as the percentage reversal of scopolamine-induced impairment (vehicle–scopolamine group = 0% inhibition, vehicle–NaCl group = 100% inhibition) in individual experiments by EGb 761^®^ and PAC. Data are shown as the means ± SEM (n = 7–14).

In the isolated PAC fraction, PACs are present mainly as dimers to hexamers and larger polymers (Kulic et al., 2022b), which would be expected to have very low oral bioavailability. To include the potential impact of low oral bioavailability of PACs in our study, we next assessed the *in vivo* activity of PACs and isolated fractions containing the established active constituents of ginkgo terpene lactones and flavone glycosides in a mouse model of scopolamine-induced memory impairment. Intraperitoneal administration of scopolamine resulted in impaired working memory of mice characterized by a significant decrease in the percentage of correct spontaneous alternations in the T-maze model compared to that in control animals (approximately 30% vs. 70% correct alternations in scopolamine-administered animals vs. controls). Pretreatment by oral gavage with donepezil (0.3 mg/kg) as a positive control largely prevented the drop in the percentage of correct alternations. Oral administration of EGb 761^®^ and isolated PACs 1 h prior to the T-maze assessment resulted in a dose-dependent attenuation of the scopolamine-induced impairment, with significant improvements at doses of 100 and 300 mg/kg for either treatment (Figures 3A, B). The administration of a fraction containing terpene lactones also partially reversed the scopolamine-induced effect at lower doses with an apparent drop in activity at the highest test dose of 30 mg/kg, suggesting an inverse U-shaped dose–response curve (Figure 3C). In contrast, a fraction containing isolated ginkgo flavones showed no activity in the test conditions (Figure 3D) despite proof of systemic exposure to the main ginkgo flavonols (Supplementary Figure S8). The potency and maximal efficacy were similar for full-extract EGb 761^®^ and PACs (EGb 761^®^ ID_50rel_ = 39 mg/kg, I_max_ = 68%; PAC ID_50rel_ = 30 mg/kg, I_max_ = 69%; Figure 4). The results suggest that a combination of terpene lactones and PACs, but not flavone glycosides, mediates the antiamnesic effects of EGb 761^®^ in our model.

#### Analysis of PAC metabolites

Metabolite profiling with LC-ESI-HRMS was conducted in intestinal content samples from rats after oral gavage of ginkgo PACs to investigate the absorption and metabolism of PACs. In total, 15 PAC-specific compounds were detected in the intestine content based on differential analysis (M01, M04, and M08–M20; Supplementary Table S2 and supplementary mass spectra of metabolites). One hour post dosing, the following primarily PAC monomers were detected: epigallocatechin, gallocatechin, epigallocatechin gallate, and gallic acid, but also myricetin (and its metabolites myricetin 3-O-glucuronide and 5′-O-methyl myricetin 3-O-glucuronide). Myricetin was previously reported to be a terminal unit of proanthocyanidin as a flavan-3-O-galloylated extender (Li et al., 2010). Six hours after dosing, phenyl-valerolactones and its precursors (M08, M09, and M12–M14) were formed, which are known to be metabolites of epigallocatechin gallate produced by intestinal bacteria (Takagaki and Nanjo, 2010). Furthermore, gallic acid was also present.

**FIGURE 5:**
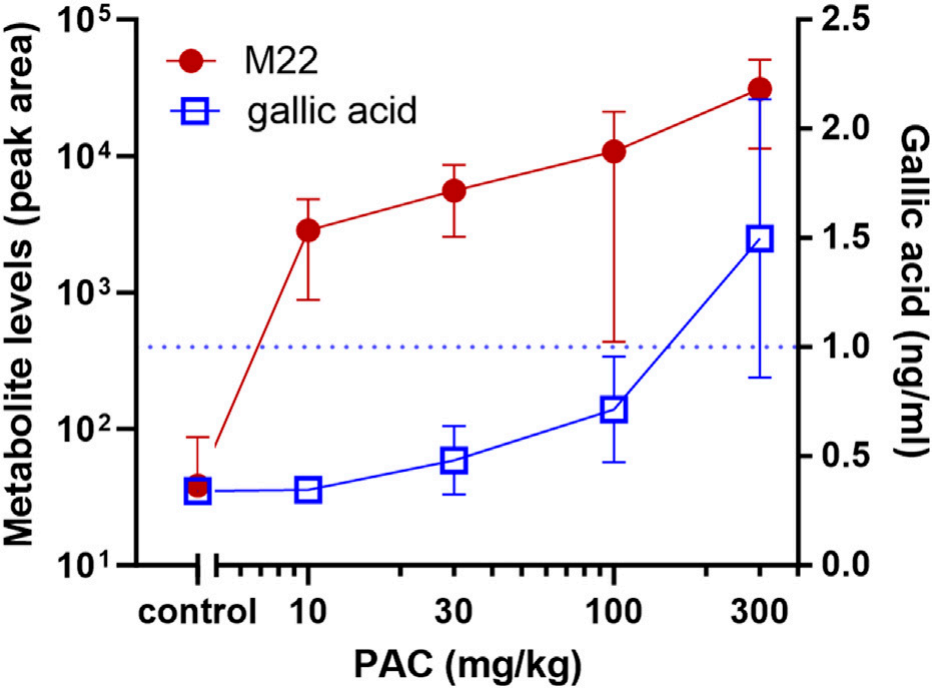
Dose dependency of PAC metabolite levels in plasma. Gallic acid (ng/mL) and peak area of M22 in mouse plasma from T-maze studies (1 h 15 min after oral administration, n = 7) dose-dependently increased with PAC dosing. PAC metabolites were identified based on untargeted analysis. Dotted line shows LLOQ of quantified analyte (gallic acid). Mass spectrometric information about metabolite M22 is included in Supplementary Table S2.

Plasma and urine samples were analyzed to study systemic bioavailability. Gallic acid phase-II metabolites were detectable in urine samples after PAC treatment of rats (M03 and M06) and in plasma samples from the mouse T-maze studies as gallic acid-O-sulfate (M02) or unchanged gallic acid (Figures 5, 7). Furthermore, methyl-myricetin (M21) and vanilla(ic acid) glucuronide were detectable (M05 and M07) in urine. One additional PAC-group-specific metabolite was detected in the plasma and urine (M22), but no reasonable PAC metabolite structure could be assigned to this molecular formula. Based on the fragmentation pattern, it is most likely a phase-II sulfate metabolite.

A sensitive targeted LC-MS/MS (TQ) method was developed to detect metabolites in plasma samples from T-maze experiments, which include PAC monomers and known metabolites, as well as metabolites identified in the untargeted analysis (Supplementary Table S2).

The analysis of the plasma samples from PAC-treated mice after finalization of the T-maze spontaneous alteration model (i.e., approximately 1 h 15 min after administration) showed a dose-dependent increase in metabolites M22 and gallic acid/M01 (Figure 5). The other 11 quantified potential PAC metabolites were either not detectable or the concentration was not different from that in the vehicle (data not shown). The latter group included endogenous metabolites such as hippuric acid.

### Kinetic correlation of the pharmacological activity and plasma levels of PAC-specific metabolites

**FIGURE 6:**
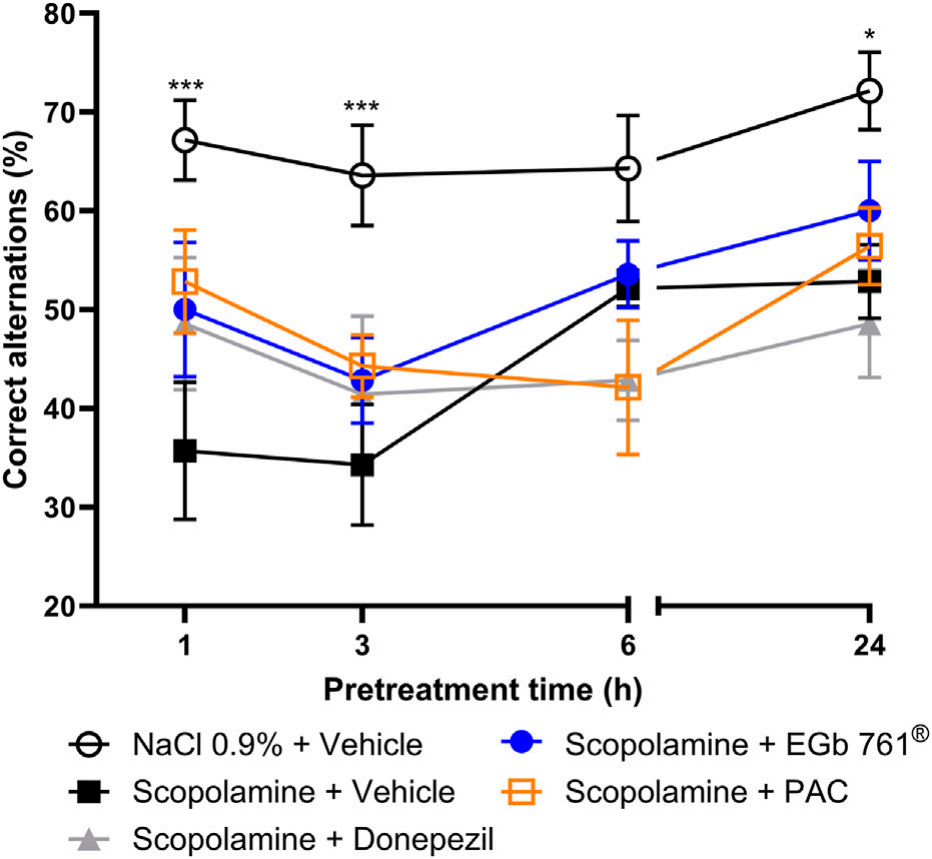
Time-dependent reversal of scopolamine-induced impairment in working memory by EGb 761^®^, PAC, and donepezil. Percentage of correct spontaneous alternations by NMRI mice in the T-maze assay (n = 20 arm entries) after impairment of working memory by i.p. injection of scopolamine (1 mg/kg) and different oral pretreatment times for EGb 761^®^ (100 mg/kg), PAC fraction (100 mg/kg), and donepezil (0.3 mg/kg). Data are shown as the means ± SEM, n = 7 per group and pretreatment time. *p < 0.05 and ***p < 0.001 vs. vehicle–scopolamine group based on two-way ANOVA with Dunnett’s test.

We next attempted to identify metabolites associated with activity by conducting a limited PK/PD-correlation analysis. To this aim, we pretreated mice orally with a single dose of PACs (100 mg/kg), EGb 761^®^ (100 mg/kg), or donepezil (0.3 mg/kg) for different time intervals (24 h, 6 h, 3 h, and 1 h) prior to the assessment of scopolamine-induced memory impairment in the T-maze model and plasma sample acquisition for PK analysis. All active test items partially reversed the scopolamine effect at 1 h pretreatment time, albeit the effects were not statistically significant. At 3 h pretreatment, the positive effects of the three active test items were already largely lost. At 6 h and 24 h pretreatment, no improvement vs. the scopolamine plus vehicle-treatment group was observed for any of the test items. However, interpretation of the 6 h and 24 h pretreatment intervals was affected by the lack of a robust scopolamine-induced impairment of the correct alterations, and hence, there was little room for improvement by the active treatments. Despite this limitation, the data suggest that for all three treatments, the maximal pharmacological effect is observed within 1 h after oral administration (Figure 6).

**FIGURE 7:**
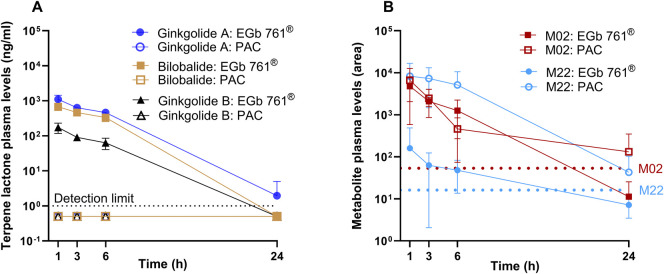
Time course of plasma levels of terpene lactones **(A)** and PAC metabolites **(B)** after administration of EGb 761^®^ (100 mg/kg) or PAC fraction (100 mg/kg). Terpene lactone levels (ginkgolide A, ginkgolide B, and bilobalide) in ng/mL plasma **(A)** and PAC metabolite (M02 and M22) peak area over time **(B)** from the PK/PD time course T-maze assay (n = 7). PAC metabolites were identified based on untargeted analysis. Dotted lines show LLOQ of quantified analytes **(A)** or background-level peak area for metabolites without standard **(B)**. Mass spectrometric information about metabolites M02/M22 is included in Supplementary Table S2.

Analysis of the plasma samples from the T-maze time-course study showed a rapid increase in the plasma concentration of terpene lactones in the EGb 761^®^ group but not in PAC-treated animals (Figure 7A). This finding indicates that the pharmacological effects of the PAC fraction were not mediated by potential terpene lactone contaminations. Plasma levels of M22 were nearly constant for 6 h, and levels of gallic acid sulfate (M02) peaked at 1 h and rapidly decreased thereafter (Figure 7B). Our limited PK/PD correlation suggests that M22 is probably not relevant for PAC activity, whereas the M02 PK profile is in accordance with the rapid loss of activity in the PAC-treated group. However, M02 was also formed after EGb 761^®^ treatment in a similar concentration range, indicating the presence of additional precursors of this metabolite in EGb 761^®^ apart from PACs.

### Impact of different PAC concentrations in finished ginkgo products on *in vivo* activity

**FIGURE 8:**
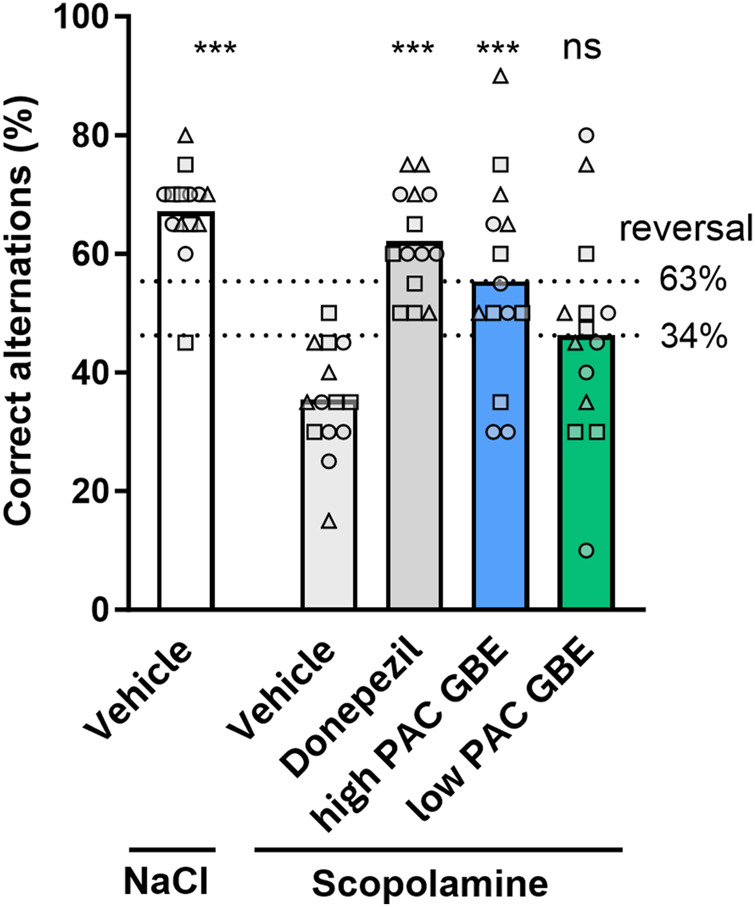
Assessment of *in vivo* antiamnesic effect of finished GBE products with either high or low PAC content (50 mg/kg, p.o.). Only the product containing a high PAC content showed statistically significant reversal of scopolamine-induced impairment in the percentage of correct spontaneous alternations. The inhibitory effect (mean percentage of reversal of scopolamine-induced impairment: 63% for high-PAC product and 34% for low-PAC product) was calculated for each individual experiment using the number of correct alternations in the vehicle + scopolamine group as 0% inhibition and the vehicle–NaCl group as 100% inhibition. Data are from three independent studies employing three different product batches with n = 4–5 animals each (indicated by different symbols). ns > 0.05, ***p < 0.001, and ****p < 0.0001, vs. scopolamine–vehicle group based on one-way ANOVA with Dunnett’s test.

To assess the relevance of different PAC contents in GBEs for pharmacological net activity at clinically relevant doses, we conducted a head-to-head comparison in our T-maze scopolamine model of two finished GBE products compliant with Ph. Eur. specifications but differing in PAC contents. The oral administration of ground tablets from three different batches of each product at a dose corresponding to 50 mg/kg of ginkgo extract produced a partial reversal of the scopolamine-induced working memory impairment. However, only the finished product with a high PAC content showed a statistically significant effect (63% reversal of the effect of scopolamine, p < 0.001), whereas the improvement induced by the low-PAC product did not reach statistical significance (34% reversal, p > 0.05) (Figure 8).

As an isolated terpene lactone fraction showed activity in the T-maze model (Figure 3C), we aimed to rule out potentially confounding effects of excipients or different PAC contents of the finished GBE products on the bioavailability of ginkgolide A, ginkgolide B, and bilobalide. Although ginkgo flavones apparently do not contribute to the activity of EGb 761^®^ in our model (Figure 3D), we additionally analyzed the main flavonol aglycone levels after acidic hydrolysis to obtain information regarding the flavone exposure as the second quantified constituent group in *G. biloba*.

**FIGURE 9:**
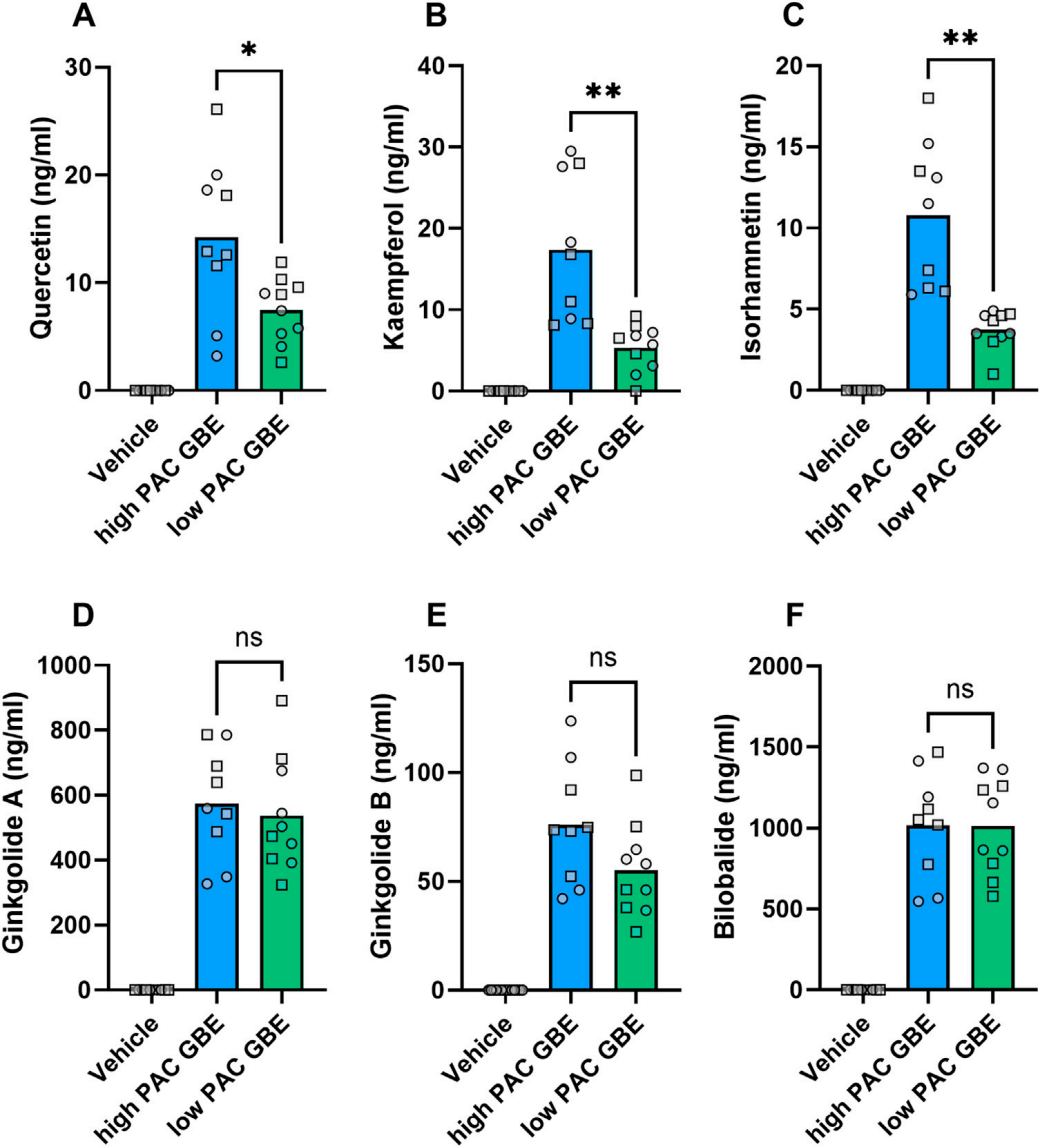
Plasma levels of flavonols and terpene lactones after administration of finished GBE products with high or low PAC content (50 mg/kg, p.o.). Higher plasma levels of flavonol aglycones (quercetin **(A)**, kaempferol **(B)**, and isorhamnetin **(C)**) but not of terpene lactones (ginkgolide A **(D)**, ginkgolide B **(E)**, and bilobalide **(F)**) in animals treated with high- vs. low-PAC GBE products. Quantitative analysis of flavonol aglycone and terpene lactone concentrations in plasma sampled immediately after assessment of working memory in the T-maze model (t = 1 h 15 min after oral administration of GBE products). Data are expressed as the means and individual values. ns > 0.05, *p 0.05, and **p < 0.01 based on two-sided unpaired t-test with Welch’s correction.

We observed significantly lower plasma flavonol aglycone levels in animals receiving the low-PAC GBE product. In contrast, no significant differences were found for plasma levels of the terpene lactones between high- and low-PAC GBE products (Figure 9).

## Discussion

*G. biloba* leaf extracts are clinically used in a range of different neurological and vascular indications. Much of the available preclinical and clinical data have been generated with the ginkgo biloba leaf extract EGb 761^®^ (Morató et al., 2024) with a specification range for the content of ginkgo flavone glycosides (22%–27%) and terpene lactones (5.4%–6.6%) that are considered to contribute to the activity of the extract. In current EU, US, and Chinese pharmacopoeias, the contents of flavone glycosides and terpene lactones are specified as either an amount range (EDQM, 2025; United States Pharmacopeial Convention, 2025) or a minimal amount (Chinese Pharmacopoeia Commission, 2020). In summary, the relative amount of specified constituents in ginkgo extracts in the regulated environment is approximately 30%, whereas the remaining 70% of the extract can vary depending on the production process of the respective extract manufacturers. The potential contribution of the unspecified constituents to the activity and safety of ginkgo extracts has not been sufficiently addressed yet, which is in part due to the complex quantitative analysis of the constituents present as oligo- and polymers.

One of the non-specified constituent classes comprises PACs, which have been reported for ginkgo leaves (Stafford et al., 1986) and ginkgo extract (Lang and Wilhelm, 1996) using different analytical methodologies. Recently, using a newly developed quantitative HPLC method, a mean PAC content of 7% was reported for the extract EGb 761^®^ (Kulic et al., 2022b), indicating that PACs are a quantitatively relevant constituent class in EGb 761^®^.

To assess whether PACs contribute to the pharmacological activity profile of EGb 761^®^, we studied Ginkgo PACs isolated from EGb 761^®^ in different *in vitro* and *in vivo* pharmacological assays.

In the rat neuronal cell line RN46A, the isolated PAC fraction showed highly potent inhibition of basal production of ROS. The potency was higher than that of the total extract EGb 761^®^ and the isolated flavone fraction, whereas a fraction containing mainly terpene lactones was essentially inactive in this assay. This suggests that PACs, in addition to the specified flavones, are likely to contribute to the antioxidant activity of EGb 761^®^. Indeed, a comparison of two commercial products containing extracts with a high PAC content (5.0%) vs. a low PAC content (0.6%) showed higher potency of the high-PAC product in our assay despite similar specified flavone content. The antioxidant activity of PACs in our cellular assay is in line with other studies that reported *in vitro* 1,1-diphenyl-2-picrylhydrazyl (DPPH) radical scavenging activity of the flavan-3-ols and proanthocyanidins isolated from *G. biloba* (Qa’dan et al., 2011). In our assay system, we assessed the test item effects on basal ROS generation but not on pathologically increased stimulated ROS production. In addition, we only used a pretreatment time of 30 min. Hence, potential effects on the induction of antioxidative enzyme systems or inhibition of induction of ROS producing enzymes would have been missed in our experimental approach. Nevertheless, direct antioxidative effects, which were probably mediated via direct scavenging of ROS, as detected in our system, would also be expected to exert beneficial effects in conditions of pathologically increased ROS production. The potential therapeutic relevance of the antioxidant effects has been assessed in cerebral ischemia–reperfusion injury models in rats. Intraperitoneal injection of PACs isolated from ginkgo or grape seeds reduced the infarct size and attenuated the markers of oxidative stress, such as malondialdehyde levels (Cao et al., 2016; Kong et al., 2017), suggesting that protective activities were at least in part mediated by antioxidative effects. Elevated ROS production is also considered relevant as a key driver of pathophysiology in neurodegenerative diseases such as Alzheimer’s disease and Parkinson’s disease. When ROS levels surpass antioxidant defenses, oxidative stress ensues—damaging neuronal membranes, disrupting enzyme function, impairing synaptic activity, and triggering neuronal death (Gogna et al., 2024).

A recently published study by Lejri et al. reported improved bioenergetics and stimulation of neurite outgrowth by PACs isolated from EGb 761^®^ (the same material as in our study) with similar efficacy as the whole extract in an *in vitro* assay in human neuroblastoma cells (Lejri et al., 2025). Their and our data indicate that PACs contribute to the pharmacological *in vitro* profile of EGb 761^®^.

The analytical characterization of the PAC fraction used in our studies indicated that PACs were present primarily as dimeric to hexameric oligomers and larger polymers with further polymerization after the isolation of the fraction in solution (Kulic et al., 2022b). PAC oligo- and polymers are expected to have very low oral bioavailability, thus questioning the predictive value of PAC efficacy data from *in vitro* and i.p. injection studies for orally administered ginkgo products such as EGb 761^®^.

To address this issue, we assessed the efficacy of EGb 761^®^ as compared to the isolated PAC fraction upon oral administration in a mouse model of pharmacologically induced memory impairment. In the T-maze model, spontaneous alternation as a readout of working memory was significantly impaired by i.p. injection of scopolamine and restored by acetylcholinesterase inhibitor donepezil. This memory impairment was dose-dependently attenuated by oral pretreatment with both EGb 761^®^ and the PAC fraction with similar potency and maximal efficacy. To the best of our knowledge, this is the first demonstration of the oral activity of ginkgo PACs in a model of scopolamine-induced working memory impairment. Our results are in agreement with Xu et al., who reported reversal of scopolamine-induced impairments in the Morris water maze and the step-down avoidance test by PACs extracted from lotus seedpod at similar doses as used in our study (50 mg/kg–150 mg/kg p.o.) (Xu et al., 2009). Beneficial treatment effects of PACs from ginkgo and other sources such as grape seeds have also been reported for animal models of cerebral ischemia–reperfusion (Cao et al., 2018; Kong et al., 2017; Yao et al., 2020) and cellular and mouse transgenic models of Alzheimer disease (Lian et al., 2016; Wang et al., 2012; Xie et al., 2014; Lee et al., 2010). Our data, thus, add to the increasing recognition of PACs as a pharmacologically active constituent group in GBEs.

Recent nonclinical studies demonstrated pro-cognitive effects of PACs from different sources upon repeated or chronic treatment in animal models. In APP/PS1 transgenic mice as a model of Alzheimer’s disease, lotus seedpod PACs administered for 5 months improved cognitive dysfunction and long-term potentiation through the stimulation of the CREB–BDNF pathway (Wang et al., 2023). In a model of thyroxin-induced aging in mice, PACs from grape seeds ameliorated learning deficits and spatial memory in aged mice, which was associated with the inhibition of acetylcholine esterase and reduction in neuroinflammation in the hippocampus (Yuan et al., 2024). In our model of scopolamine-induced memory impairment, we assessed acute effects of a single oral PAC administration. Our time-course study demonstrated the highest activity at 1 h post administration, suggesting that mechanisms acting via neuroplasticity or the inhibition of inflammation are probably of little relevance for the observed efficacy. Mechanistically, scopolamine affects cognition by interference with acetylcholine signaling via muscarinic receptor antagonism. Hence, treatments that increase acetylcholine levels to displace scopolamine from the muscarinic receptor should attenuate scopolamine-induced amnesia in our T-maze model. Indeed, acetylcholine esterase inhibitor donepezil reversed the scopolamine-induced memory impairment in our model. We observed antiamnesic effects with EGb 761^®^, PACs, and a terpene lactone fraction devoid of relevant antioxidative activity. In contrast, no antiamnesic effect was observed upon oral treatment with a flavone glycoside-enriched fraction despite having good antioxidative activity *in vitro* and the demonstration of systemic exposure. This apparent lack of correlation between *in vitro* antioxidative activity and *in vivo* antiamnesic activity suggested that the antiamnesic activity depended on a mechanism independent of the suppression of ROS production. We speculated that the acute treatment effects of EGb 761^®^ and PACs could be mediated by the inhibition of acetylcholine esterase in our model. Indeed, using enzymatic *in vitro* assays, we observed a concentration-dependent acetylcholine esterase inhibition by EGb 761^®^, the isolated PAC, and flavone fractions (Supplementary Figure S6). This is in accordance with data from lotus seedpod procyanidins and a proanthocyanidin-rich grape seed extract, which were also shown to act as acetylcholine esterase inhibitors *in vitro* (Xu et al., 2009; Hasbal-Celikok et al., 2024). However, PACs are well known for causing protein precipitation, leading to non-specific enzyme inhibition under *in vitro* conditions. Therefore, *in vitro* activities may not necessarily be relevant for oral administration. To assess whether *in vivo* treatment effects were mediated via the inhibition of acetylcholine esterase activity or modification of acetylcholine levels in general, we conducted *ex vivo* measurements of acetylcholine levels and acetylcholine esterase activity (AChE) in the plasma from the scopolamine T-maze studies. We encountered some limitations to this approach as samples had to be strongly diluted to fall into the measurable activity range of the enzymatic assay, and thus, potential inhibitory concentrations of the test items in the *in vivo* condition could have been lost by dilution. Using our experimental conditions, we did not find any relevant changes in acetylcholine levels and AChE activity in the plasma samples for any treatment, including positive control donepezil (Supplementary Figures S7A, B). Therefore, our experimental approach was not sufficiently sensitive to assess the effects on acetylcholine metabolizing enzymes.

We aimed to identify the individual compounds or metabolites that were associated with the observed pharmacological activity of the isolated PACs. Therefore, we first conducted an untargeted LC-HRMS/MS analysis from samples after the oral administration of a high dose (300 mg/kg) of EGb 761^®^ and PACs to rats. Using intestinal content samples, we detected PAC monomers, and at the 6 h time point, previously described intestinal microbiota metabolites such as phenyl-γ-valerolactones were also detected. This indicates that the metabolization of ginkgo PACs generally occurred via similar pathways as for PACs from other sources. At 1 hour post administration of PACs, gallic acid (metabolites) was detected in plasma formed probably due to the fast hydrolysis of epigallocatechin gallate. Similar concentrations of gallic acid (metabolites) were found in the EGb 761^®^ group despite the administration of a 20-fold lower overall PAC dose in the whole extract group compared to that in the PAC fraction-treated animals (Figure 7B). One explanation for this finding could be the presence of additional precursor molecules of gallic acid (metabolites) in EGb 761^®^ independent of PACs. Alternatively, PAC absorption could have been improved by the matrix effects of the whole extract. Using different pretreatment times prior to scopolamine challenge and T-maze assessment, we found that the pharmacodynamic activity was most prominent at 1 h post administration, whereas it was decreased at 3 h and was not detectable any more at 6 h and 24 h. The different time patterns of pharmacodynamic activity and pharmacokinetic profile strongly suggest that the observed pharmacological effect is not caused by microbiota-generated metabolites of PACs, as these are formed at later time points. We observed one additional metabolite M22 in plasma and urine that seemed to be primarily influenced by PAC administration. Although the time course of gallic acid plasma levels showed a rapid decrease from 1 h to 6 h, which is in accordance with the observed pharmacodynamic activity, levels of M22 remained largely constant for 6 h, suggesting that M22 was probably not relevant for the reversal of scopolamine-induced memory impairment. The assessment of plasma levels of terpene lactones showed the maximal levels at 1 h with subsequent decline in EGb 761^®^-treated animals. For the PAC-treated animals, no relevant concentrations of terpene lactones were found, confirming that the activity in the model was not mediated by potential terpene lactones impurities in the isolated PAC fraction. Except for gallic acid (metabolites), no other metabolites with a structural relationship to PACs were detectable with LC-(HR)MS/MS in plasma 1 h post treatment, albeit the pharmacodynamic activity was most prominent at this time point. This might be caused by the limited chemical space that is captured with sufficiently high sensitivity with this particular analytical method in complex matrices or due to loss during sample preparation. For instance, the detection of methyl-myricetin in the urine of PAC-treated rats indicates that additional metabolites were present systemically but were not detectable in the plasma due to sensitivity limitations. The analysis with complementary analytical methods would be needed to identify compounds beyond the limits of a generic untargeted LC-(HR)MS/MS and a targeted LC-MS/MS of known PAC metabolites. This was beyond the scope of this study.

In our scopolamine T-maze protocol, the maximal efficacy was achieved at 100 mg/kg for both EGb 761^®^ and PACs. For EGb 761^®^, this dose represents approximately two-fold of the clinically approved dose of 240 mg per day (i.e., 4 mg/kg/day) after dose normalization by body surface (Reagan-Shaw et al., 2008). For PACs, the 100 mg/kg dose (human equivalent dose of 8 mg/kg) required to achieve a significant effect in our model is far above the dose administered at the clinically approved dose of EGb 761^®^, with a content of 5%–7% PAC (0.2 mg/kg–0.28 mg/kg PACs/day). However, EGb 761^®^ also contains terpene lactones and flavone glycosides as known specified active constituents that are likely to contribute to the overall activity of EGb 761^®^. Consequently, small effects by clinically relevant PAC doses could still potentially contribute to the overall activity of EGb 761^®^ although no significant effect was observed with the employed animal number and the resultant limited statistical power of the study. In addition, our analytical studies indicated possible polymerization of PACs following isolation (Kulic et al., 2022b), suggesting that native and less polymerized PACs present in the EGb 761^®^ matrix that were not submitted to fractionation could potentially have higher oral bioavailability than the isolated fraction. To assess the contribution of native PACs to the activity of GBEs at clinically relevant doses, we conducted a head-to-head comparison of two finished GBE products differing in their PAC content. The product containing a GBE with a high PAC content (5.0%) produced a significant reversal of 63% of the scopolamine-induced impairment at the dose of 50 mg/kg (corresponding to 4 mg/kg/day in humans after allometric normalization). In contrast, the low-PAC product (0.6%) showed only a nonsignificant reduction by 34% at the same nominal extract dose. Both products contained GBE produced according to the Ph. Eur. and thus, by definition, had comparable levels of terpene lactones and flavone glycosides.

In the T-maze experiments, a fraction isolated from EGb 761^®^ containing terpene lactones successfully reversed scopolamine-induced amnesia (Figure 3C). To accurately interpret the observed differences in activity between high- and low-PAC GBEs, it was, therefore, essential to eliminate any potential interference of the different excipients in the finished products on the bioavailability of terpene lactones. We quantified the levels of ginkgolide A, ginkgolide B, and bilobalide in plasma samples collected immediately after assessing the reversal of scopolamine-induced impairment of working memory in the T-maze model. No significant differences in the plasma levels were found between the two treatments (Figure 9), indicating that the higher activity of the high-PAC GBE product was not mediated by effects on the bioavailability of ginkgolides A and B or bilobalide. Unexpectedly, we found significantly higher plasma levels of the main ginkgo flavonols after treatment with the high-PAC vs. the low-PAC GBE product. Both were registered medicinal products complying with the Ph. Eur. specifications. Therefore, largely identical ginkgo flavone glycoside contents can be assumed. Hence, either product-specific excipients or the higher PAC content apparently improved the bioavailability of flavonols in the high-PAC GBE product. We did not have access to capsules with excipients only of the commercial products or the extract without excipient in the case of the low-PAC product to assess the excipient effects on the bioavailability of PACs. Therefore, the cause of the differences in flavonol exposure between the products remains elusive. Several studies in the literature indicated the activity of flavonoids, including flavonols quercetin (Olayinka et al., 2022) and isorhamnetin (Ishola et al., 2019), on scopolamine-induced memory impairment when using repeated administration over several days. In contrast, in our model employing a single acute oral administration, ginkgo flavonols administered in an isolated fraction from EGb 761^®^ devoid of terpene lactones and PACs did not show any improvement of scopolamine-impaired memory (Figure 3D) despite producing equal or higher plasma exposure levels as observed with the finished GBE products (Supplementary Figure S8). Therefore, differences in flavonol exposure are unlikely to contribute to the higher *in vivo* activity of the high-PAC GBE product in the applied experimental setting.

As no significant differences were observed in the terpene lactone levels, which could have affected the interpretation of the activity data, it seems plausible that the difference in PAC content (quantity or quality) was at least partially responsible for the differences in the activity between the two GBE products in our model when tested at clinically relevant doses.

In conclusion, our data show that PACs isolated from the *G. biloba* extract EGb 761^®^ exert antioxidative activity in a cellular model and reverse scopolamine-induced memory impairment upon oral administration. Comparison of finished ginkgo products with different PAC contents indicated higher activity of the high-PAC ginkgo extract in both *in vitro* and *in vivo* assays. Our study provides initial evidence that in addition to terpene lactones and flavone glycosides, the PAC content could be relevant for the activity profile of EGb 761^®^ and potentially of other ginkgo extracts with a high PAC content. If confirmed by further nonclinical and clinical studies, this could be an important finding, as the PAC content is not specified at the regulatory level to date.

## Data Availability

The original contributions presented in the study are included in the article/Supplementary Material; further inquiries can be directed to the corresponding author.
