# Supplementary material for: Proanthocyanidins from Ginkgo biloba extract EGb 761® exert antioxidative activity in vitro and antiamnesic activity in vivo
Source: Front Pharmacol. 2025 Oct 27;16:1673248. doi: 10.3389/fphar.2025.1673248 (PMC12597941; doi:10.3389/fphar.2025.1673248)
Supplement: Supplementary file 1 [file DataSheet1.pdf]

Supplementary Material

Supplementary Data

Materials and Methods

Analysis of PAC content in commercial Ginkgo extract herbal medicinal products

PAC content in commercial Ginkgo extract herbal medicinal products was analyzed as described previously (Germer et al., 2024). In brief, film-coated tablets from each batch of Tebonin® konzent® 240 mg and Kaveri® 120 mg were milled to fine powder without removing coating. For each sample, an amount equivalent to 100 mg Ginkgo extract was dissolved in hydrolysis solution, sonicated, stirred, and centrifuged. The resulting solutions were hydrolyzed in a boiling water bath for 45 minutes, cooled, and tempered to room temperature. Clear solutions were transferred to HPLC vials for analysis without further processing.

HPLC analysis was conducted using a Thermo UltiMate 3000® system with a Kromasil® C18 column and a water/methanol gradient. Detection was at 530 nm, with a 20-minute run time. Delphinidin and cyanidin were quantified using hydrolyzed procyanidin B2 standards, with delphinidin calculated as cyanidin. Pelargonidin was excluded due to low signal. A response factor of 2.12 was applied to convert procyanidin B2 results to PAC content.

Ethynyl-2'-dioxyuridine (EdU) proliferation assay

RN46A cells were seeded (100.000 cells/well, 6 well plate) and cultured overnight. Treatment occurred for 48 h with DMSO 0.1 %, a three concentrations of EGb 761®, and fractions containing either PACs, terpene lactones or flavone glycosides, respectively (1, 10, 100 µg/ml). Cell staining was performed using the EdU Flow Cytometry Kit 488 (BCK-FC-488-50, Sigma). Briefly, EdU was added for 1 h before harvesting the cells and followed by the EdU-detection procedure and 7AAD staining. Analysis was performed at a Novocyte flow cytometer using the NovoExpress software.

Cell Viability Assay

RN46A cells were seeded in a Poly-L-lysine (P4832, Sigma Aldrich) coated 96-well plates at a density of 5,000 cells per well in 100 µl of culture medium. Cells were treated for 24 h with either 0.1 % DMSO as a solvent control, a serial dilution of EGb 761® (0.03 µg/ml -100 µg/ml) or defined concentrations of EGb 761® and PACs (25 µg/ml EGb 761® or 2 µg/ml PAC). Cell viability was assessed using the CellTiter 96® AQueous One Solution (G3580, Promega). The reagent was thawed in a 37°C water bath for 20 minutes prior to use. Subsequently, 20 µl of the reagent was added to each well, and the plates were incubated at 37 °C for 2 hours. Absorbance was measured at 490 nm using a SpectraMax M Multimode Microplate Reader (Molecular Devices).

Assessment of quenching effects of test items on DCF fluorescence in the absence of cells

Serial dilutions of extract and fractions were prepared in absence of phenol red in Dulbecco's Modified Eagle's Medium/Nutrient Mixture F-12 Ham (D-6434, Sigma-Aldrich) to yield a final concentration of 0.03 µg/ml - 100 µg/ml. Thereof, 50 µl were mixed with 50 µl of a 500 µM DCF solution (35848, Sigma-Aldrich) in Corning® 96-well Flat Clear Bottom Black Polystyrene TC-treated Microplates (3603, Corning). After a 30 min incubation time the fluorescence was measured at 524 nm in a SpectraMax M Multimode Microplate Reader (Molecular Devices).

Acetylcholine Esterase Assay

To evaluate direct inhibitory effects on acetylcholine esterase (AChE) activity we adapted a protocol from Ellman et al (Ellman et al., 1961). The reaction mix was prepared in DPBS (D8662, Sigma Aldrich) containing a final concentration of 1 mM DTNB (5`-Dithiobis(2-nitrobenzoic acid)) (D218200, Sigma Aldrich) and 100 µM Acetylcholine Iodide (A5751, Sigma Aldrich). AChE from *Electrophorus electricus* (C3389, Sigma Aldrich) was dissolved 20 mM Tris HCl buffer, pH 7.5 (8382, Merck) and was applied in the assay in a final concentration of 0.4 U/ml. Serial dilutions of EGb 761®, its purified fractions PACs, terpene lactones or flavones (0.03 µg/ml, 0.1 µg/ml, 0.3 µg/ml, 1 µg/ml, 3 µg/ml, 10 µg/ml, 30 µg/ml, 100 µg/ml) were tested. Reactions were set up in a clear 96-well microplate. Briefly, 45 µl AChE solution were mixed with 5 µl test substance or control (0.1 % DMSO) and the reaction was started by the addition of 150 µl of the DTNB- and Acetylcholine Iodide-containing reaction mix. For the evaluation of AChE activity in *ex vivo* samples, plasma from animals tested in the T maze model was diluted 1:40 in phosphate buffered saline (D-8662, Sigma-Aldrich) and 50 µl of each sample was loaded per well in a 96-well microplate followed by the addition of 150 µl reactions mix. Absorbance of the yellowish reaction product 5-thio-2-nitrobenzoate was measured using a SpectraMax M Multimode Microplate Reader (Molecular Devices) at 410 nm and percentage of AChE activity was calculated.

Quantification of Acetylcholine in plasma

The Amplex® Red Acetylcholine/Acetylcholinesterase Kit (A12217, Invitrogen) was applied to assess acetylcholine (ACh) concentration in plasma samples from animals used in the T maze model. Plasma was diluted 1:20 in reaction buffer, ACh standard curve and reaction mix containing 400 µM Amplex® Red reagent, 2 U/mL HRP, 0.2 U/mL choline oxidase and 1 U/mL acetylcholinesterase were prepared as described in the manufacturer's instructions. Each 100 µl per standard or sample were applied per well in a clear flat-bottom 96 well plate and 100 µl reaction mix was added, followed by an incubation for 30 min at room temperature. Fluorescence signals (Ex 560 nm, Em 590 nm) were measured using a SpectraMax M Multimode Microplate Reader (Molecular Devices), background fluorescence was subtracted and of ACh concentration was calculated.

Flavonol aglycone quantification in plasma

0.2 mg/l stock solution of isorhamnetin and 1 mg/l quercetin and kaempferol were prepared in methanol. Quercetin-d3 was used as internal standard. Working solutions were prepared by diluting in milli-q water. The calibration range for quercetin and isorhamnetin was 1-300 ng/ml plasma and for kaempferol 2.5- 300 ng/ml plasma. Quality control samples (QCs) were prepared by spiking desired levels of flavone to blank plasma.

Briefly, 300 µl of plasma were combined with 10 µl 20 % ascorbic acid, 20 µl 0.58 M acetic acid, 30 µl internal standard solution and 80 µl enzyme solution (500 Units β-Glucuronidase from bovine liver) thoroughly mixed and kept 1 h at 37 °C. The mixture was diluted with 90 µl 2 % formic acid, vortexed, an aliquot of 342 µl transferred to a conditioned RP-SPE-vessel (Bond Elut Plexa,30 mg, Agilent) and the solution adsorbed on the solid phase by slight suction. The column was washed with 500 µl 1 % formic acid in water. Analytes were finally eluted 2 times with each of 300 µl methanol (MeOH) /acetonitirile (ACN) (1:1; v/v) with 1% formic acid into a vessel containing 25 µl 0.1 % ascorbic acid in MeOH. The combined organic solvents were removed by evaporation at 30 °C. Subsequently, the residue was taken up in 300 µl Eluent A. Finally, 10 µl of the filtrate were injected for HPLC-MS/MS analysis (Eluent A: ACN/water (2.5%:97.5%; v/v) + 0.1% formic acid; Eluent B: ACN/water (97.5%:2.5%; v/v) + 0.1% formic acid).

The LC instrument setup consisted of an Agilent 1290 Infinity II HPLC and an Agilent 6470A Triple Quadrupole MS/MS. Chromatographic separation was performed with a Water Acquity UPLC high-strength silica (HSS) T3 1.8 µm (2.1 mm × 150 mm) column (Waters, Wilmslow, UK). The gradient conditions of the mobile phase were as follows: 0–1 min 0 % B; 1–5 min 0–30 % B; 5–12 min, 30–100 % B; 12-15 min 100 % B, 15.00-15.1 100-0 %B, 15.1-20 min 0 % B. Target analytes were analyzed in ESI- ionization mode with multiple reaction monitoring (MRM) using tandem mass spectroscopy (MS/MS) with the following transitions as quantifier: isorhamnetin (315 to 300), quercetin (301 to 151) and kaempferol (285 to 185). The source conditions were as follows: gas temperature of 210°C, flow rate of 4 l min^{-1} , and capillary voltage of 2000 V(−), Nozzle Voltage 0 V, Sheath gas temperature 400°C and sheath gas flow 12 l min^{-1}.

Data processing was conducted with mass hunter quantitative analysis 10.1.

Terpene lactone quantification in plasma

0.1 mg/l stock solution of ginkgolide A, ginkgolide B and bilobalide were prepared in methanol. Ginkgolide B ethyl ether was used as internal standard. Working solutions were prepared by diluting in 0.1 M HCl. The calibration range for ginkgolide A and bilobalide was 2.4-200 ng/ml plasma and for ginkgolide B 5- 100 ng/ml plasma. Quality control samples (QCs) were prepared by spiking desired levels of terpene lactones to blank plasma.

Briefly, 250 µl of 1:10 diluted sample or blank plasma for QC or calibration standard (30 µl plasma sample and 270 µl 0.1 M HCI,) was combined with 50 µl internal standard. Then 250 µl of the mixture was transferred to a ChemElut-LE-vessel (Agilent, 75530260), the solution was adsorbed on the solid phase by slight suction and left for 10 min for equilibration. The adsorbed terpene lactones were eluted 3 times with 270 µl tert.-butylmethylether (Merck, 1.01845.1000) and the combined organic phases were removed by evaporation at 40° C and dried over night at room temperature with P_2O_5 (Merck, 1.00543.2800) Subsequently the residue was dissolved in 200 µl N-Methyl-N-trimethylsilyl-trifluoroacetamide (MSTFA, Thermo scientific, TS48914) and 1µl of the solution was injected for GC-MS/MS analysis.

An Agilent 7890B GC (Agilent, Santa Clara, CA) coupled to an Agilent 7000D triple-quadrupole MS (Agilent, Santa Clara, CA) was used to analyze all samples. A PAL3 Serie II RSI 120 autosampler (CTC Analytics AG, Zwingen, Switzerland) were mounted on the GC system. The analytes were separated chromatographically using the following GC ramping program: hold at 120°C for 1 min; ramp at 20°C/min to 320°C; hold for 7.3 min. The total GC run time was 17 min. Target analytes were analyzed in negative ion chemical ionization with multiple reaction monitoring (MRM) mode using tandem mass spectroscopy (MS/MS) with the following transitions as quantifier: ginkgolide A (390 to 237), ginkgolide B (460 to 387), bilobalide (380 to 246) and ginkgolide B ethyl ether (416 to 387).

Data processing was conducted with mass hunter quantitative analysis 10.1.

Supplementary Figures and Tables

Supplementary Figures

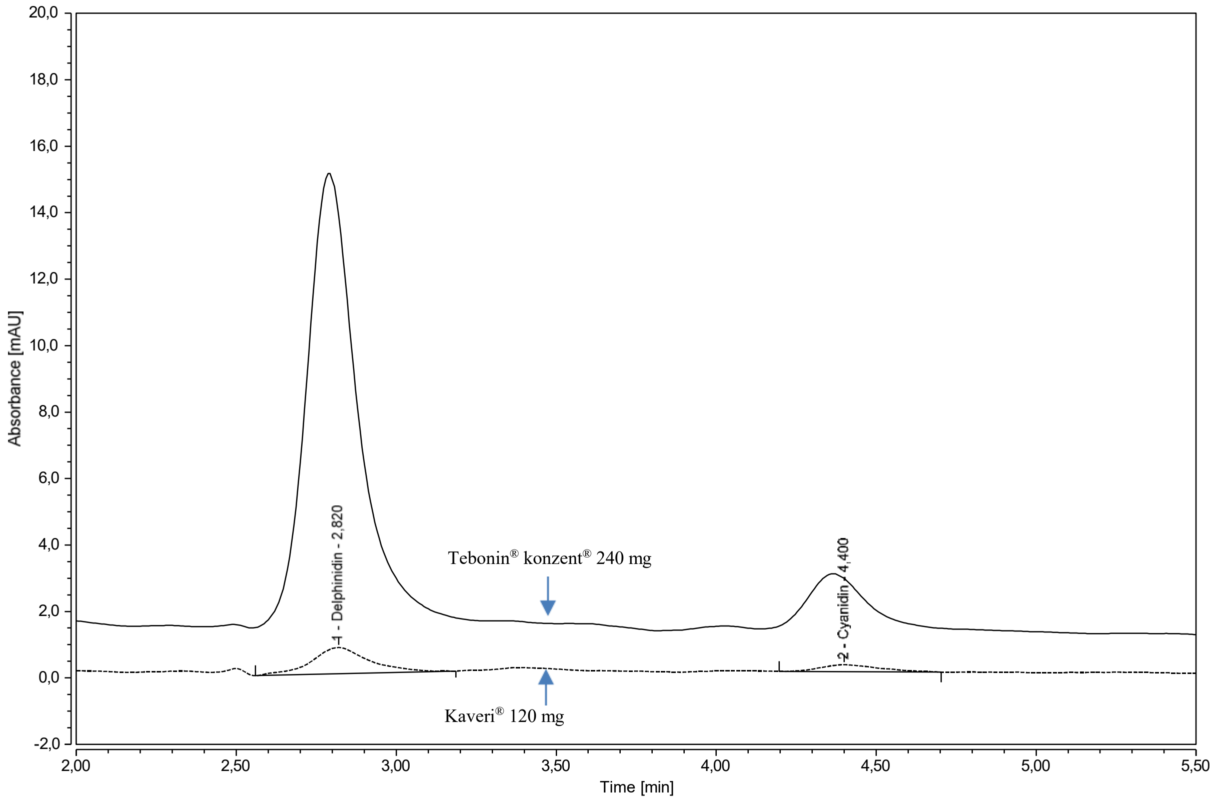

Supplementary Figure S1: Overlay of exemplary HPLC chromatographs for the high PAC (Tebonin® konzent® 240 mg, batch 0080320) and low PAC (Kaveri® 120 mg, batch 181120) commercial Ginkgo extract products. The exemplary chromatograph shows the quantitative differences in delphinidin and cyanidin content between both products.

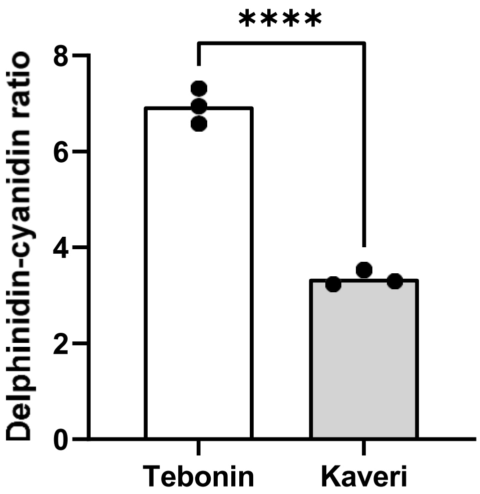

Supplementary Figure S2: Higher delphinidin-cyanidin ratio in the high PAC GBE product. Analysis of three different batches of the high PAC (Tebonin® konzent® 240 mg) and low PAC (Kaveri® 120 mg) Ginkgo extract commercial products demonstrates significant differences in the ratio of delphinidin to cyanidin between the two extracts (Delphinidin mean ± SD: Cyanidin Tebonin®: 6.94 ± 0.37:1 ; Kaveri®: 3.35 ± 0.16:1). Statistical analysis was done with two-sided unpaired t-test, **** $p < 0.0001$)

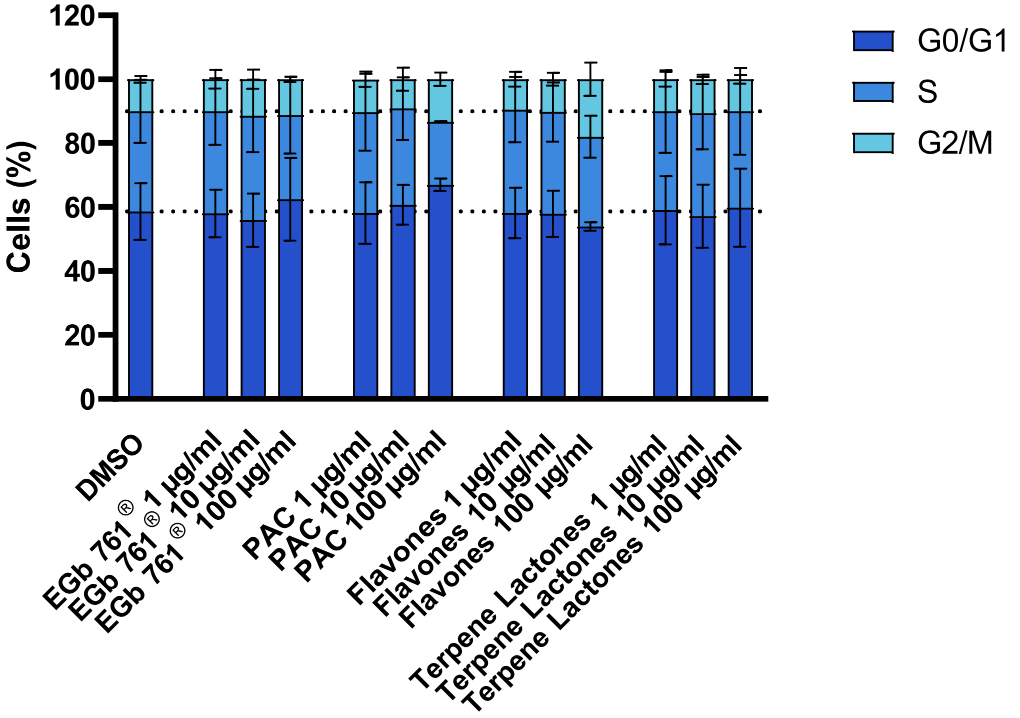

Supplementary Figure S3: EGb 761® and isolated fractions do not affect cell cycle of RN46A cells. No significant changes in cell cycle progression were observed by treating RN46A cells for 48 h with DMSO 0.1 %, or three concentrations of EGb 761®, and fractions containing either PACs, terpene lactones or flavone glycosides (1, 10, 100 µg/ml). Two independent experiments were conducted and data are presented as means ± SD.

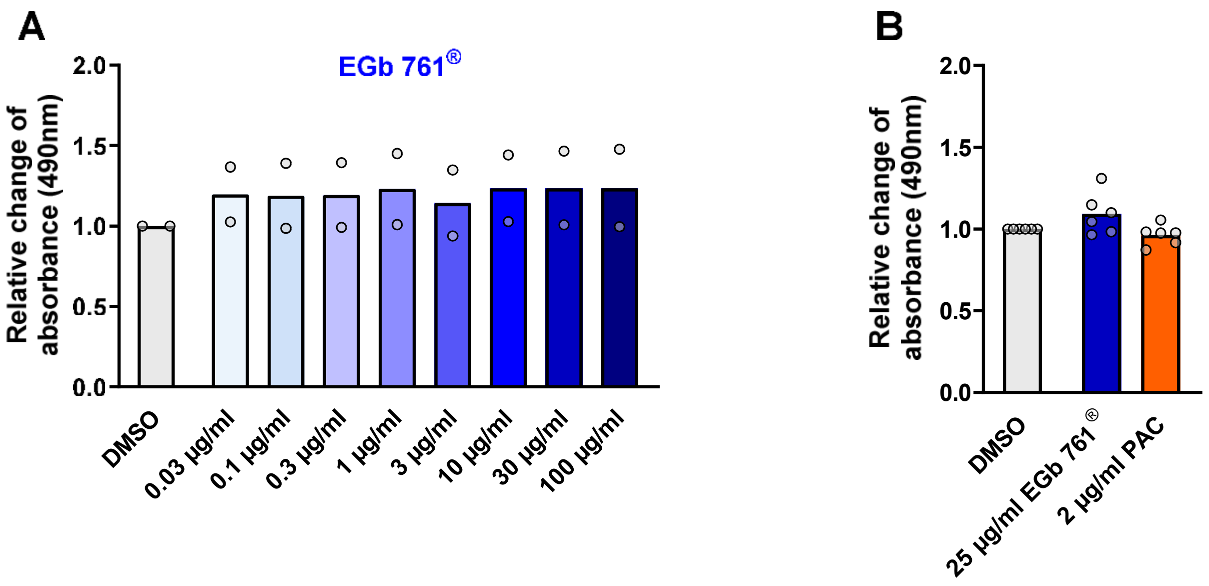

Supplementary figure S4: EGb 761® and PACs do not negatively affect the viability of RN46A cells. Viability of RN46A cells was not biased by a treatment for 24 h with either 0.1 % DMSO as a solvent control, a serial dilution of EGb 761® (0.03 µg/ml -100 µg/ml) or at defined concentrations of EGb 761® and PACs (25 µg/ml EGb 761® or 2 µg/ml PAC). Data show means of 2 (A) or 6 (B) independent experiments with individual values.

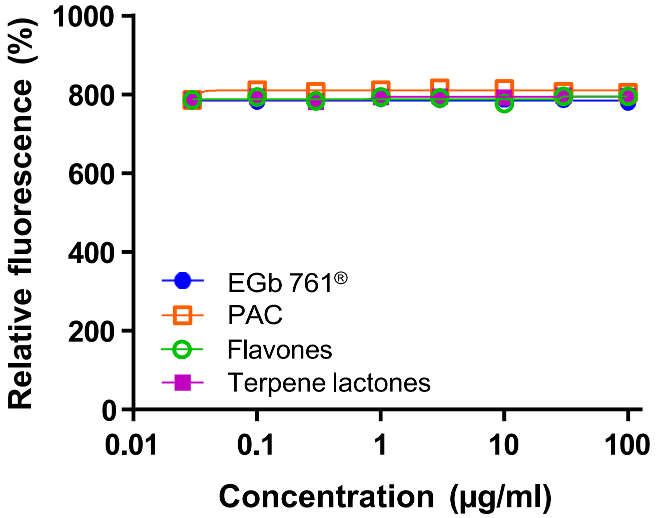

Supplementary figure S5: EGb 761® isolated fractions containing PACs, terpene lactones and flavone glycosides do not affect DCF fluorescence in the absence of cells. Serial dilutions of extract and fractions (0.03 µg/ml - 100 µg/ml) were tested to assess potential quenching effects on the fluorescence of DCF which is the fluorescent product in the cellular reactive oxygen species (ROS) assay when applying DCFDA / H2DCFDA to cells. Fluorescence of DCF was not affected. Data are shown as means of n = 2 wells.

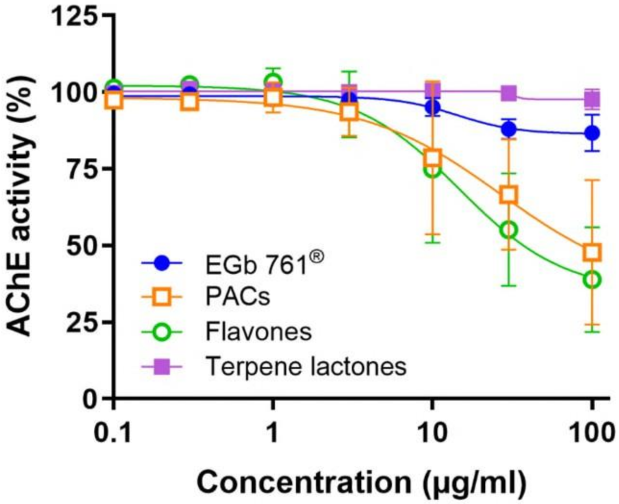

Supplementary Figure S6: Flavones and PACs but not terpene lactones inhibit acetylcholine esterase activity when added *in vitro* in an enzymatic assay system. Means and SD of n = 4.

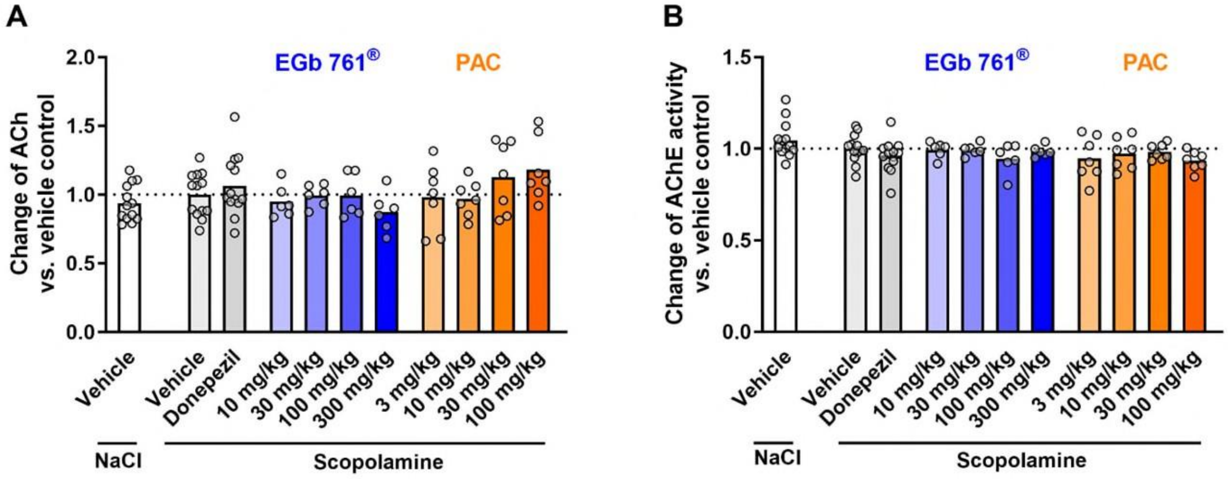

Supplementary Figure S7: Effect of EGb 761® and PACs on the AChE activity and ACh level in plasma.

Acetylcholine (ACh) levels and acetylcholine esterase (AChE) activity were analyzed in plasma collected from mice directly after assessment of working memory in the T maze model shown in figure 3A and B (t = 1 h 15 min after oral administration). Only marginal effects could be shown for PAC-treated animals but none upon EGb 761® treatment on changes of ACh plasma levels (A) and AChE activity (B). Data is presented as means and individual values of relative changes to vehicle- and scopolamine-treated animals (n = 6 - 13 animals).

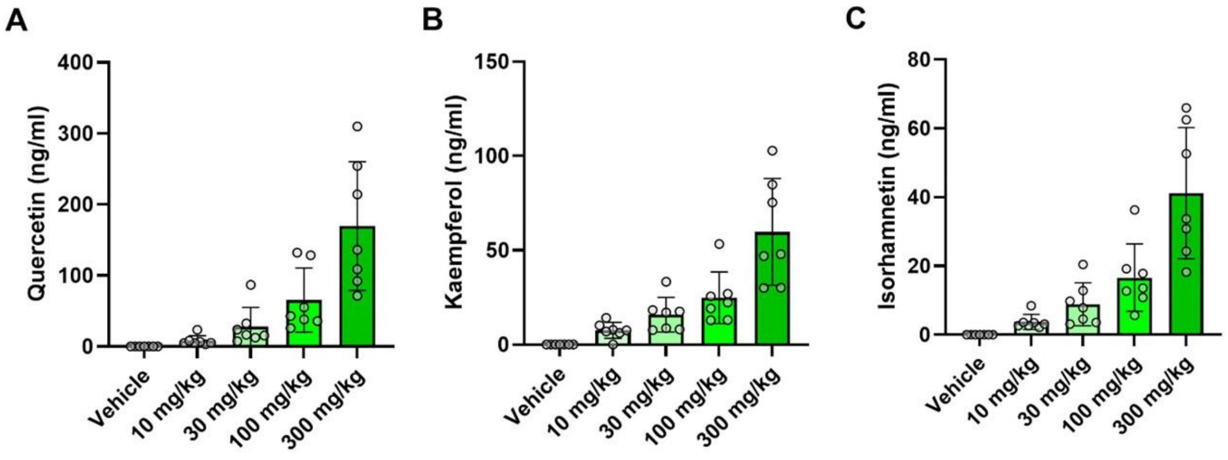

Supplementary Figure S8: Oral administration of a Ginkgo flavone-glycoside enriched fraction leads to dose-dependent increases in plasma flavonol levels.

Dose-dependent increase in flavonol plasma levels in animals treated with a Ginkgo flavone glycoside-enriched fraction and subjected to analysis of spontaneous alternation in the T-maze model (results from T-maze study in Figure 3D). Plasma samples were obtained at 1 h 15 min after oral administration of a Ginkgo flavone glycoside enriched fraction immediately after testing in the T-maze spontaneous alternation model. Plasma was submitted to acidic hydrolysis prior to quantification of flavonol aglycone levels of quercetin (A), kaempferol (B) and isorhamnetin (C). Data are shown as means ± SD and individual animal sample values (n = 7).

Supplementary Tables

Supplementary Table 1: Mass transitions for potential PAC metabolites

Analyte	Q1 mass (*m/z*)	Q3 mass (*m/z*)	Frag(V)	CE(V)	RT (min)	Polarity	LLOQ (ng/mL)
3- (2-Hydroxyphenyl) propionic acid	165.1	121.1	95	13	14.19	Negative	1
3- (2-Hydroxyphenyl)propionic acid	165.1	106	95	25	14.19	Negative	1
3-(3-Hydroxy-4-methoxyphenyl)propionic-d3 Acid	198.1	136.1	130	13	13.96	Negative	IS
3-(3-Hydroxy-4-methoxyphenyl)propionic-d3 Acid	198.1	135	130	25	13.96	Negative	IS
4-hydroxybenzoic acid	137	93.1	70	13	8.48	Negative	1
4-hydroxybenzoic acid	137	65.2	70	37	8.48	Negative	1
4-Hydroxybenzoic Acid-d4	141.1	97.1	90	13	8.38	Negative	IS
Apigenin-d5	274.1	120.1	150	41	17.51	Negative	IS
Apigenin-d5	274.1	119	150	41	17.51	Negative	IS
Bilobalid	325.1	251.1	105	5	13.99	Negative	1
Bilobalid	325.1	163.1	105	17	13.99	Negative	1
Dihydro Isoferulic Acid-d3 3-O--D-Glucuronide	398.1	222	125	13	12.3	Positive	IS
Epigallocatechin	307.1	139	125	13	9.67	Positive	5
Epigallocatechin	305.1	125	140	21	9.67	Negative	5
Ferulic acid	193.1	178	95	9	13.85	Negative	1
Ferulic acid	193.1	134	95	17	13.85	Negative	1
Gallic acid	169	125	115	12	2.96	Negative	1
Gallic acid	169	79	115	25	2.96	Negative	1
Gallic Acid-d2	171	127	110	13	2.88	Negative	IS
Gallic Acid-d2	171	81	110	25	2.88	Negative	IS
Gallocatechin	307.1	139	115	17	6.8	Positive	5
Gallocatechin	305.1	125	135	24	6.8	Negative	5
Ginkgolid A	453.1	407.1	110	9	16.4	Negative	1
Ginkgolid A	453.1	351.1	110	17	16.4	Negative	1
Ginkgolid B	423.1	367.1	130	13	16.39	Negative	1
Ginkgolid B	423.1	125	130	33	16.39	Negative	1
Guaiacol--D-glucopyranoside d3	334.1	334	95	0	10.75	Negative	IS
Hippuric acid	180.1	105	70	17	9.8	Positive	1
Hippuric acid	180.1	77.1	70	41	9.8	Positive	1
Isoferulic Acid-d3 3-O-Sulfate	276	196	125	17	12.4	Negative	IS
Isoferulic Acid-d3 3-O-Sulfate	276	178	125	21	12.4	Negative	IS
M02	249	169	100	10	4.3	Negative	no quantification
M02	249	125	100	10	4.3	Negative	no quantification
M06	263	183	100	10	6.8	Negative	no quantification

**Analyte**	**Q1 mass (*m/z*)**	**Q3 mass (*m/z*)**	**Frag(V)**	**CE(V)**	**RT (min)**	**Polarity**	**LLOQ (ng/mL)**
M06	263	168	100	10	6.8	Negative	no quantification
M22	277	197	130	20	18.7	Negative	no quantification
M22	277	182	130	34	18.7	Negative	no quantification
o-Hydroxyhipuric acid	196.1	121	70	13	12.9	Positive	1
o-Hydroxyhipuric acid	196.1	65.1	70	45	12.9	Positive	1
p-Coumaric acid	163	119.1	85	16	13.1	Negative	1
p-Coumaric acid	163	93.1	85	37	13.1	Negative	1
p-Coumaric-d6 Acid	169.1	125.1	100	13	12.95	Negative	IS
p-Coumaric-d6 Acid	169.1	97.1	100	37	12.95	Negative	IS
Protocatechuic acid	153	109	100	12	6.11	Negative	1
Protocatechuic acid	153	108	100	29	6.11	Negative	1
5-[(3,4-dihydroxyphenyl)methyl]oxolan-2-one	207.1	163	145	16	12.59	Negative	5
5-[(3,4-dihydroxyphenyl)methyl]oxolan-2-one	207.1	122.1	145	21	12.59	Negative	5

Supplementary Table 2: Identified metabolites based on untargeted analysis with LC-HRMS

ID	Name	RT (min)	Formula	Monoisotopic mass	Measured mass	Delta mass (ppm)	Main Fragments Ions	Identification Level [a]	Compartment	Time points
**M01**	Gallic acid	1.9	C7H6O5	170.02152	170.02151	-0.05	125.0240	Level 1	Intestine, Plasma[c]	6h<br>1h
**M02**	Gallic acid-O-sulphate	4.9	C7H6O8S	249.97834	249.9783	0.20	169.0152	Level 3	Plasma[c]	
**M03**	Gallic acid glucuronide	5.011	C13 H14 O11	346.0536	346.0535	-0.39	No MS2 spectra	Level 4	Urine	1h
**M04**	Gallocatechin	5.9	C15 H14 O7	306.07395	306.0737	-0.8	125.02398<br>179.03556	Level 1	Intestine	1h, 6h
**M05**	Vanillic acid glucuronide	6.215	C14 H16 O10	344.0744	344.0742	-0.39	113.0245<br>152.0198<br>175.0255	Level 3	Urine	1h,6h
**M06**	Methylgallic acid-O-sulphate	6.5	C8 H8 O8 S	263.994	263.9939	-0.53	124.01675<br>168.06999<br>183.03067	Level 3	Urine	1h,6h
**M07**	Vanillin glucuronide	7.975	C14 H16 O9	328.0794	328.0792	-0.63	113.0245<br>136.01680<br>151.0454<br>175.0253	Level 3	Urine	1h,6h
**M08**	5-(3',4',5'-trihydroxyphenyl)-gamma-Valerolactone	8.3	C11 H12 O5	224.06847	224.0685	0.12	138.03201<br>179.07126	Level 3	Intestine	6h
**M09**	1-(3‘,4‘,5‘-trihydroxyphenyl)-3-(2‘‘,4‘‘,6‘‘-trihydroxyphenyl)propan-2-ol	9.4	C15 H16 O7	308.08960	308.08937	-0.77	139.0399<br>167.0436	Level 3	Intestine	6h
**M10**	Epigallocatechin	9.5	C15 H14 O7	306.07395	306.0737	-0.84	125.0239<br>179.0346	Level 1	Intestine	1h, 6h
**M11**	Catechin	9.7	C15 H14 O6	290.07904	290.079	-0.12	245.08208	Level 1	Intestine	6h
**M12**	5-(3‘,5‘-Dihydroxyphenyl)-γ-Valerolactone	10.8	C11 H12 O4	208.07356	208.07356	-0.02	123.0447<br>163.0757	Level 3	Intestine	6h

ID	Name	RT (min)	Formula	Monoisotopic mass	Measured mass	Delta mass (ppm)	Main Fragments Ions	Identification Level [a]	Compartment	Time points
**M13**	5-(3‘,4‘-Dihydroxyphenyl)-γ-Valerolactone	12.2	C11 H12 O4	208.07356	208.07356	-0.03	163.07629<br>122.03711	Level 1	Intestine	6h
**M14**	1-(3′,4′-dihydroxyphenyl)-3-(2″,4″,6″-trihydroxyphenyl)propan-2-ol	12.2	C15 H16 O6	292.09469	292.0947	-0.11	123.04467<br>247.09747	Level 2 [b]	Intestine	6h
**M15**	Epigallocatechin gallate	12.5	C22 H18 O11	458.0849	458.0848	-0.23	125.02389<br>169.01395<br>305.06717	Level 1	Intestine	1h
**M16**		12.5	C10 H10 O11	306.0223	306.0231	2.44	169.01395	No Identification, (gallic acid metabolite)	Intestine	1h
**M17**	(2*Z*)-5-(3,4-dihydroxy-5-methoxyphenyl)-4,5-dihydroxy-3-phenylpent-2-enoic acid	12.5	C18 H18 O7	346.10525	346.1046	-1.8	205.05060<br>283.09756<br>327.08728	Level 3	Intestine	6h
**M18**	Myricetin 3-O-glucuronide	12.8	C21 H18 O14	494.0697	494.0697	0.07	151.0034<br>317.02954	Level 3	Intestine	1h
**M19**	5'-O-methyl Myricetin 3-O-glucuronide	14.0	C22 H20 O14	508.0853	508.0853	-0.08	316.02197<br>331.04690	Level 3	Intestine	1h
**M20**	Myricetin	15.3	C15 H10 O8	318.0376	318.0372	-1.04	137.00248<br>151.0059<br>178.99829	Level 1	Intestine	1h, 6h
**M21**	5-O-Methylmyricetin	16.5	C16 H12 O8	332.0532	332.0532	-0.20	316.0204	Level 3	Urine	6h
**M22**		19.7	C14H14O4S	278.06128	278.06123	-0.19	197.09787	No Identification, sulfate metabolite	Plasma	1h, 6h

a: According to Schymanski et al 2014
b: Literature spectra from Takagaki and Nanjo 2012
c: Metabolites detected in mice plasma

Mass spectra of metabolites

M01: Gallic acid

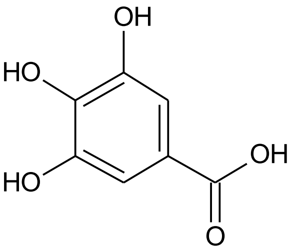

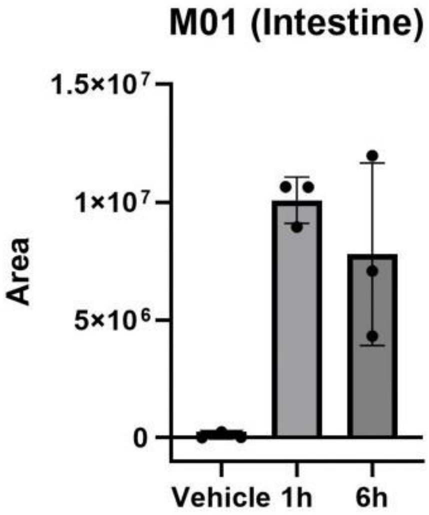

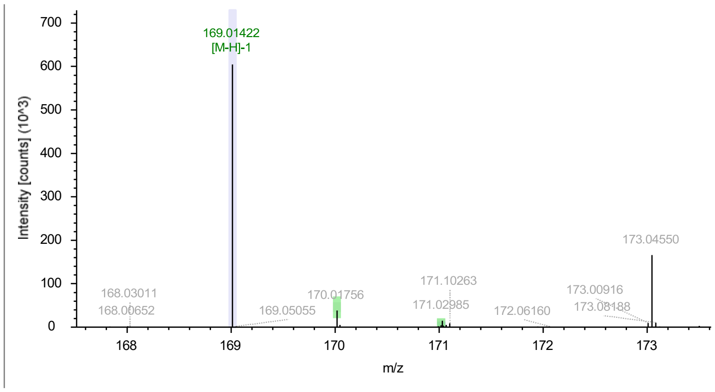

Negative full scan; RT 1.9 min

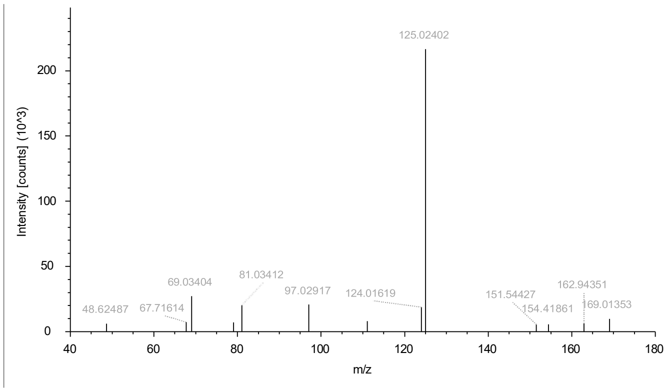

RT 1.9 MS2, FTMS(-), HCD, 169.0142 @ 30.50.70

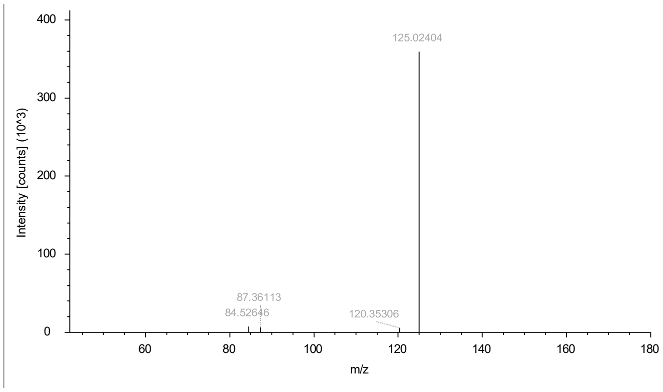

RT 1.9 MS2, FTMS(-), CID, 169.0142 @ 30

M02 Gallic acid-O-sulphate

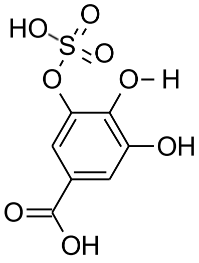

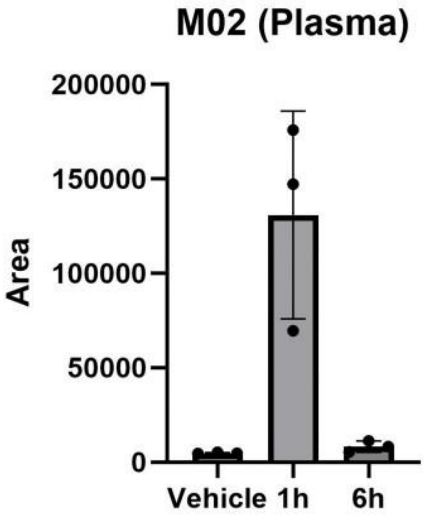

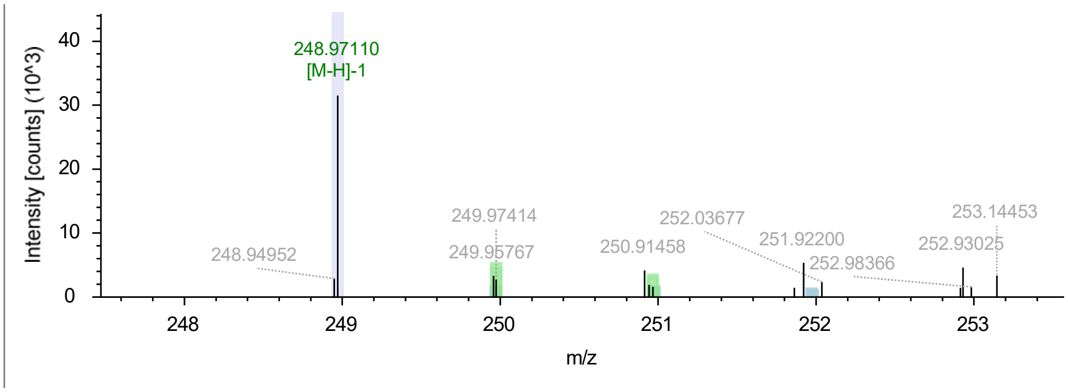

Negative full scan; RT 4.9 min

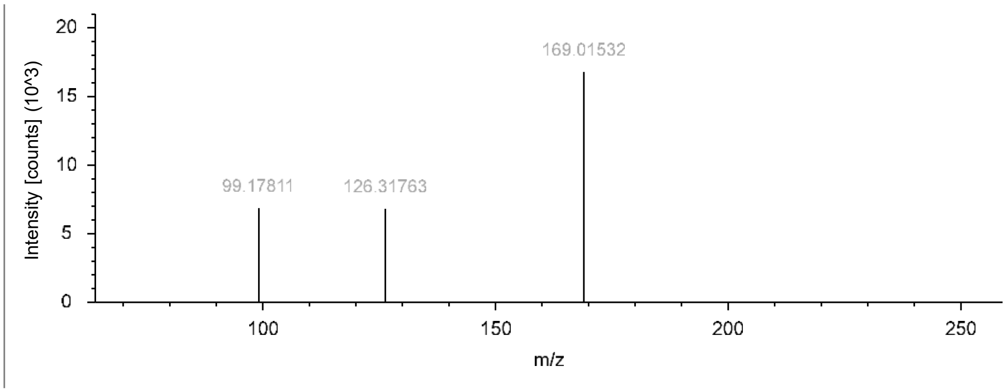

RT 4.9 MS2, FTMS(-), CID, 248.971 @ 30

M03 Gallic acid glucuronide

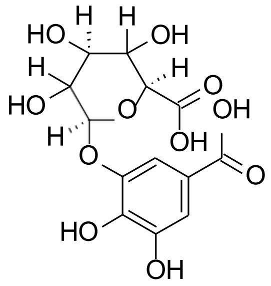

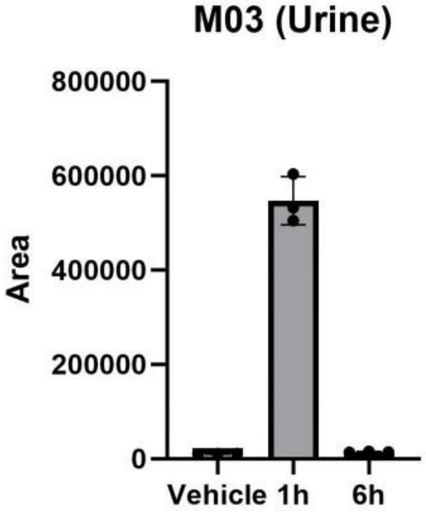

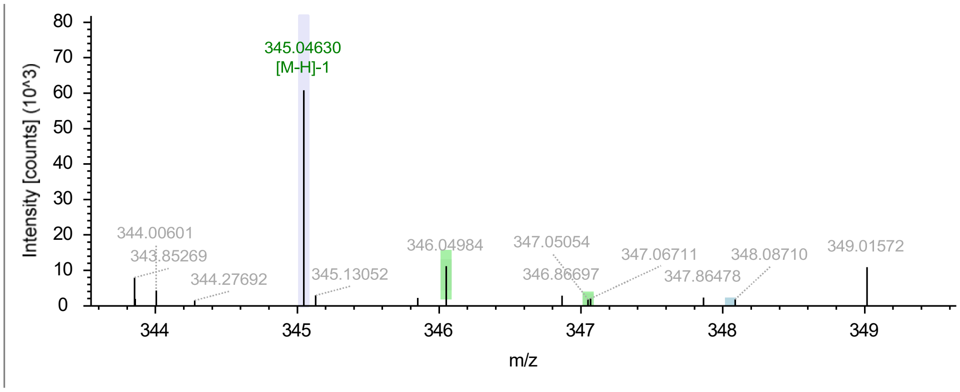

Negative full scan; RT 5.0 min

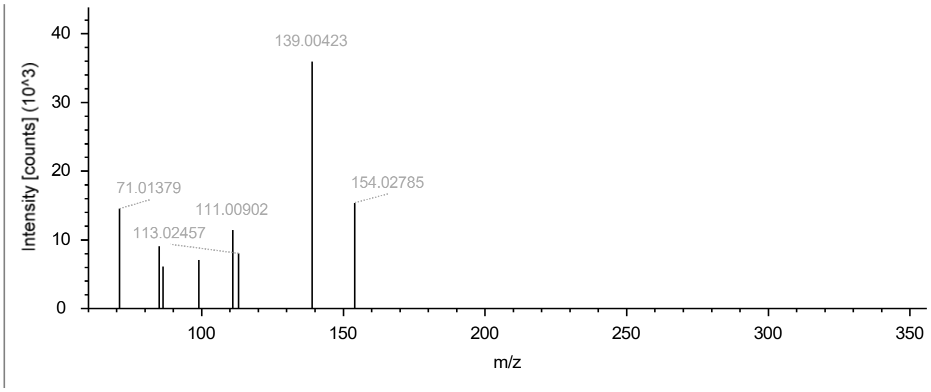

RT 5.0 MS2, FTMS(-), HCD, 345.0826 @ 30,50,70

M04 Gallocatechin

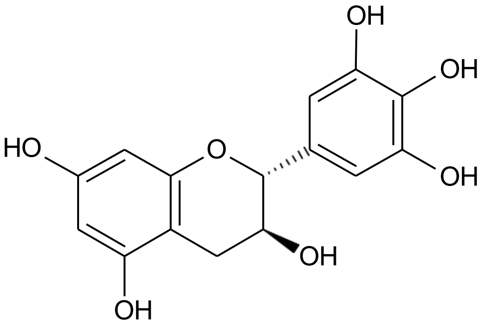

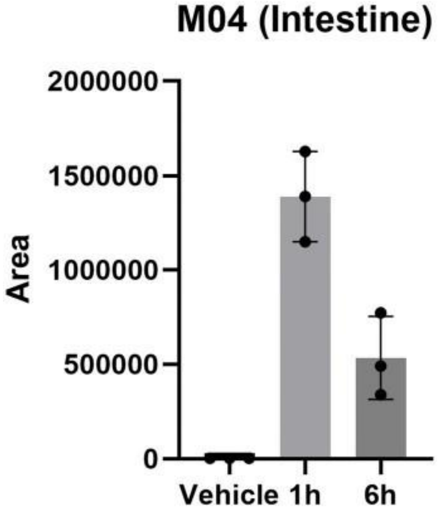

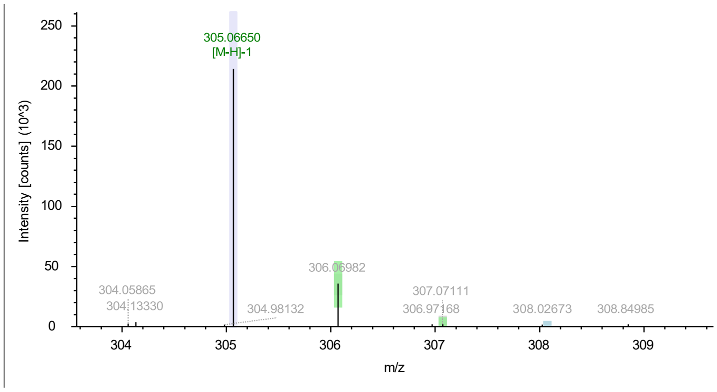

Negative full scan; RT 5.9 min

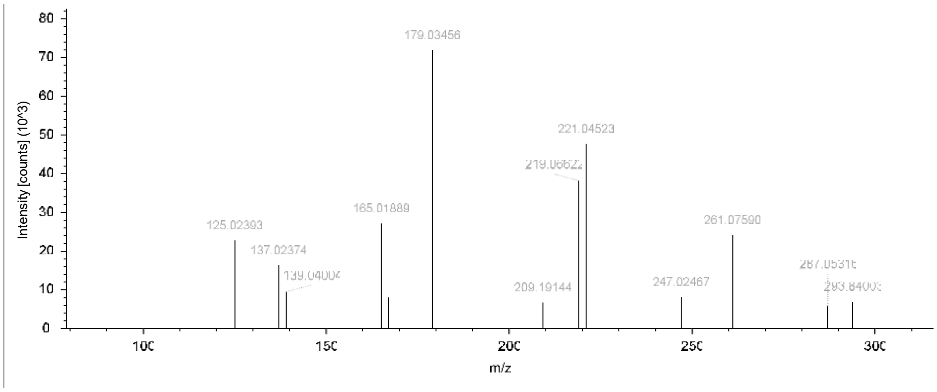

RT 5.9 MS2, FTMS(-), CID, 305.0665 @ 30

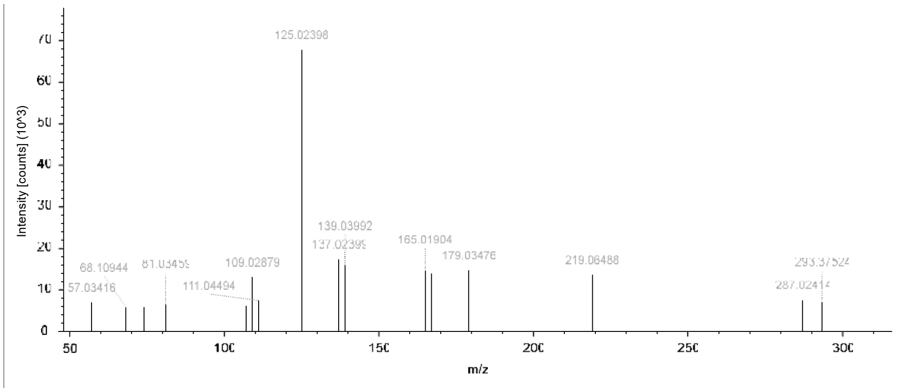

RT 5.9 MS2, FTMS(-), HCD, 305.0665 @ 30,50,70

M05: Vanillic acid 4-O-glucuronide

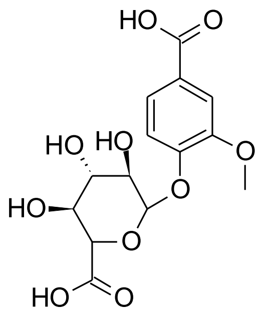

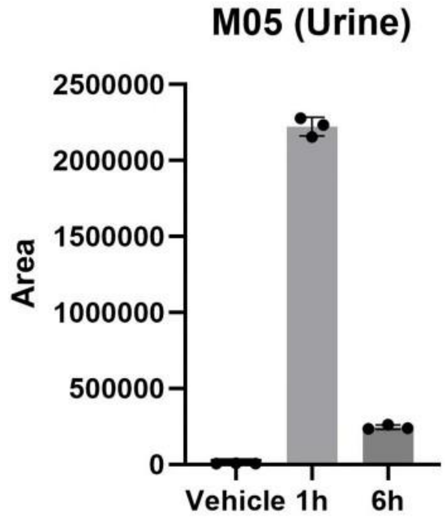

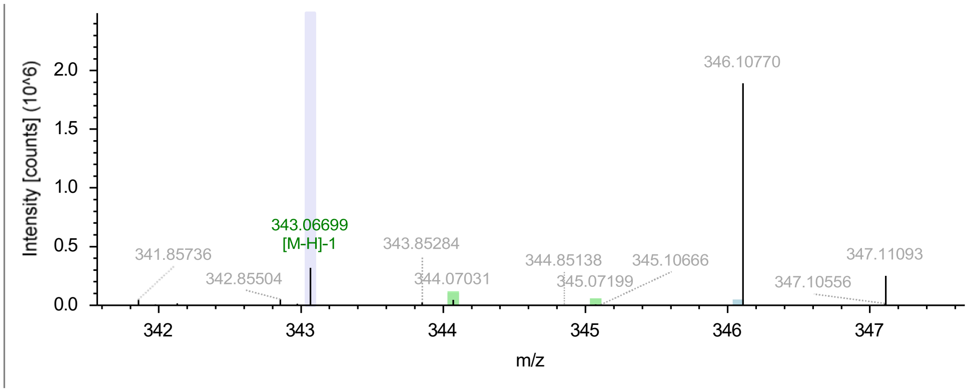

Negative full scan; RT 6.2 min

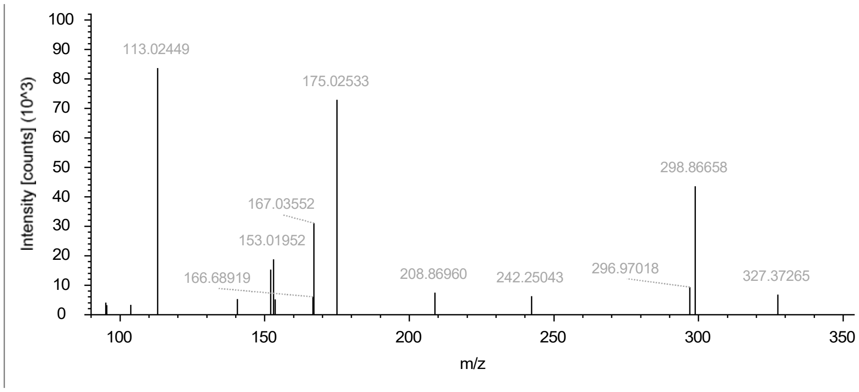

RT 6.2 MS2, FTMS(-), CID, 343.0670 @ 30,50,70

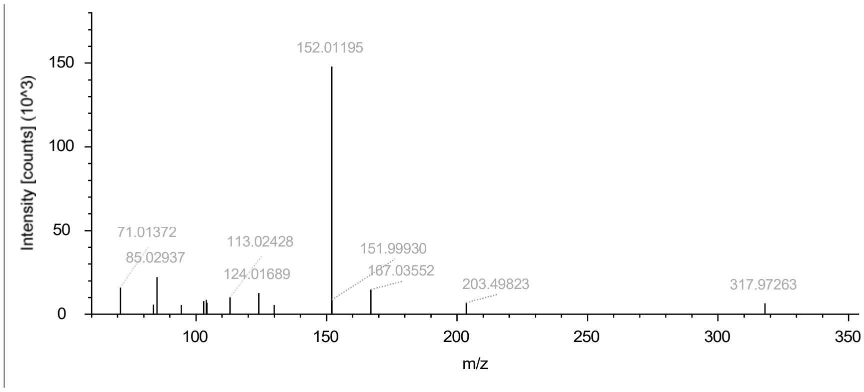

RT 6.2 MS2, FTMS(-), CID, 343.0670 @ 30

M06 Methylgallic acid-O-sulphate:

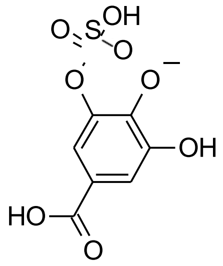

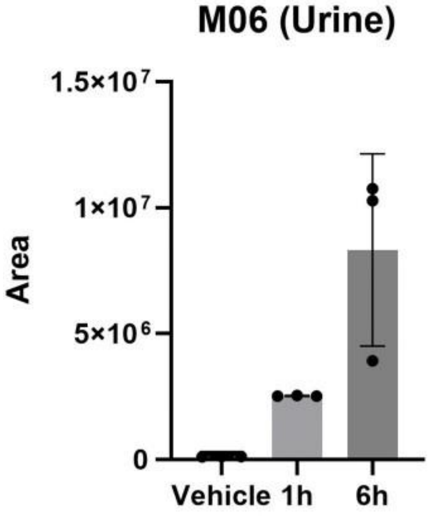

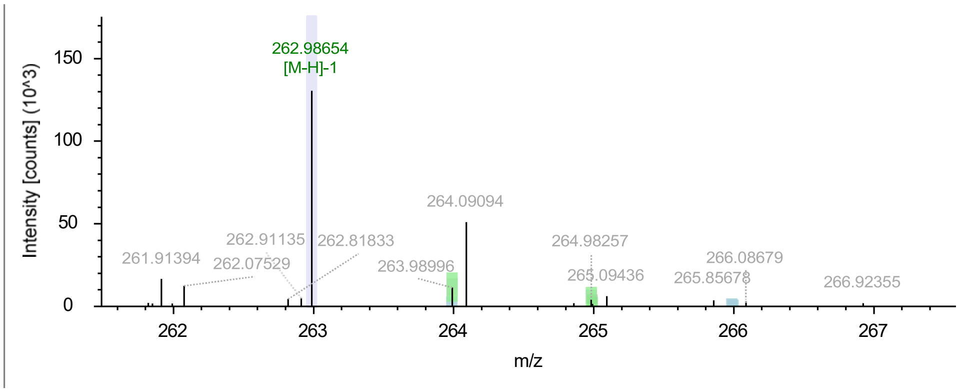

Negative full scan; RT 6.5 min

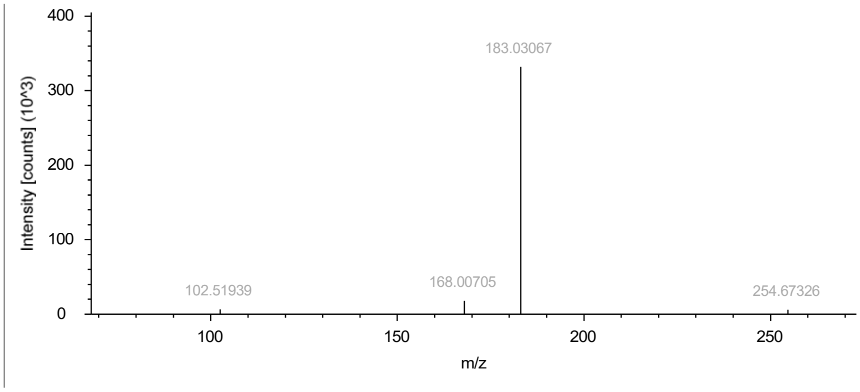

RT 6.5 MS2, FTMS(-), CID, 262.9865 @ 30

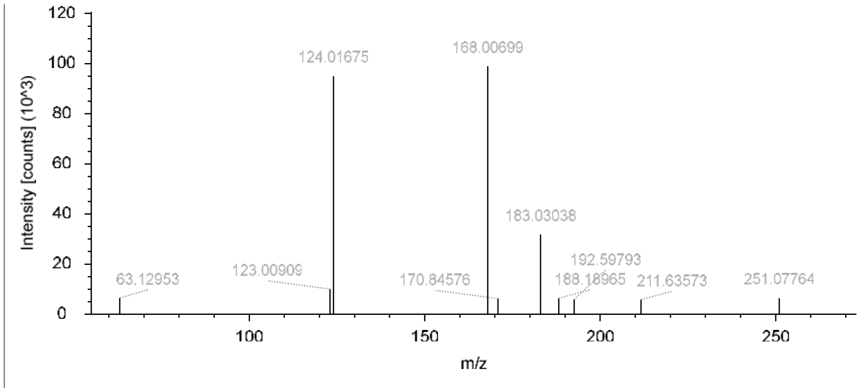

RT 6.5 MS2, FTMS(-), HCD, 262.9865 @ 30,50,70

M07 Vanillin glucuronide

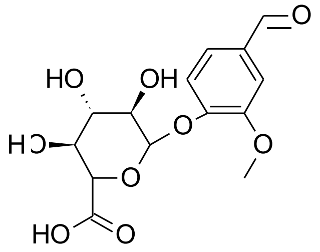

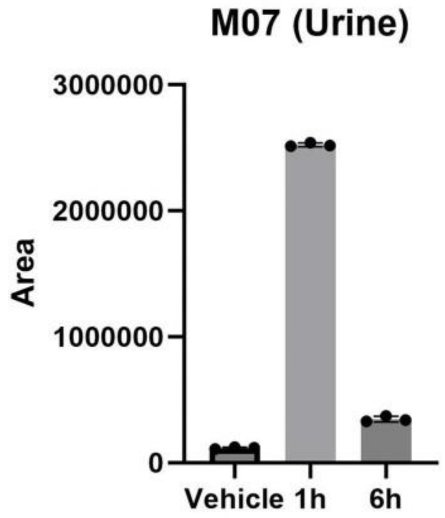

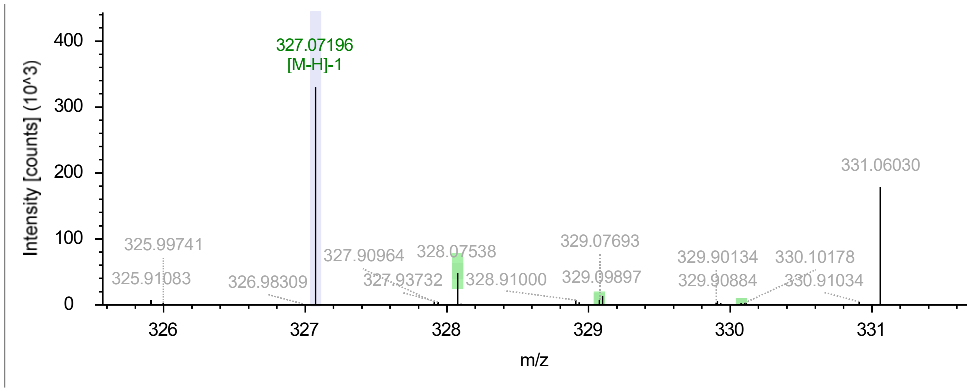

Negative full scan; RT 7.9 min

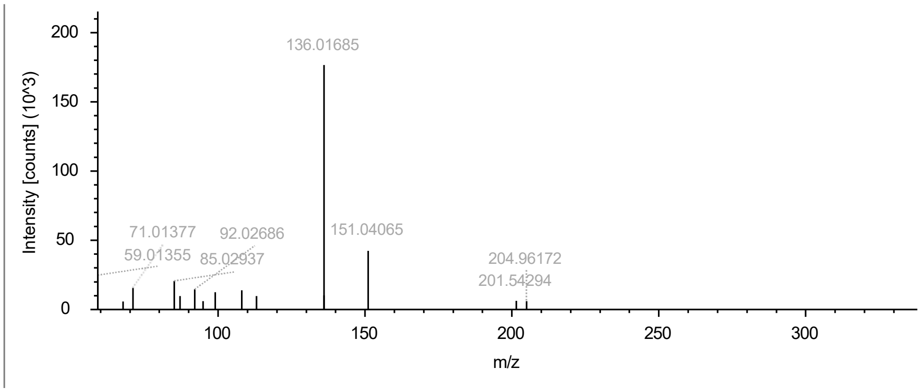

RT 7.9 MS2, FTMS(-), HCD, 327.0720 @ 30,50,70

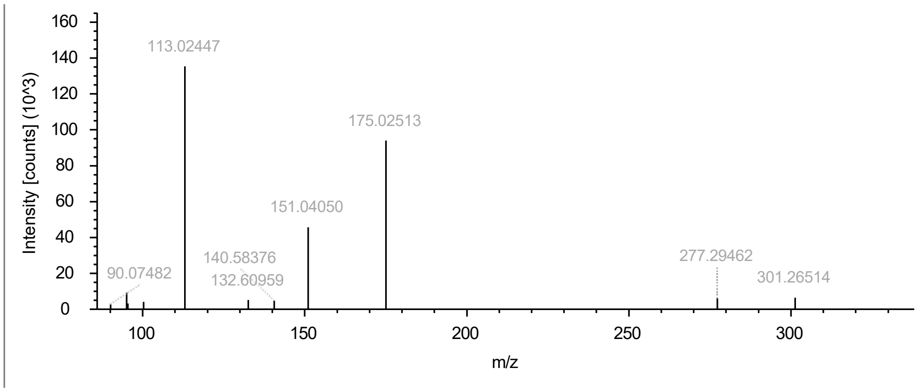

RT 7.9 MS2, FTMS(-), CID, 327.0720 @ 30

M08: 5-(3',4',5'-trihydroxyphenyl)- γ -valerolactone

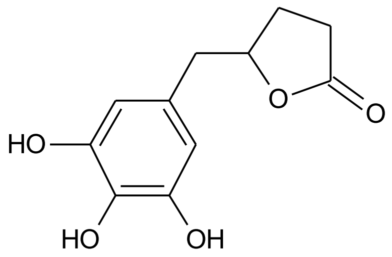

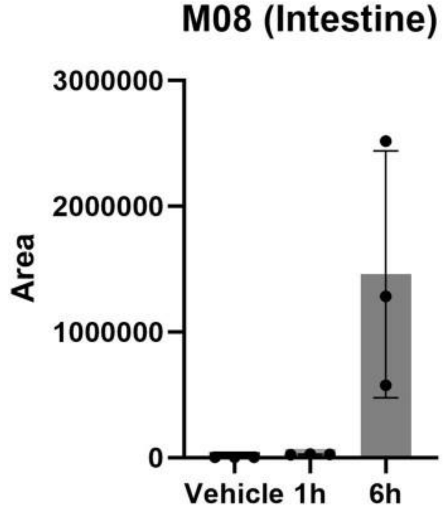

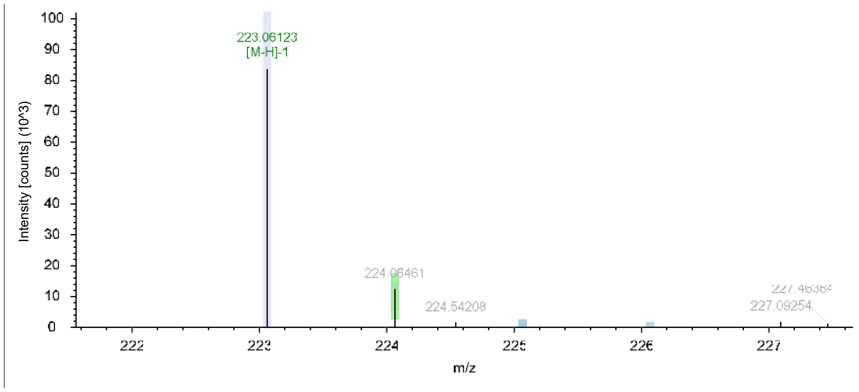

Negative full scan; RT 8.2 min

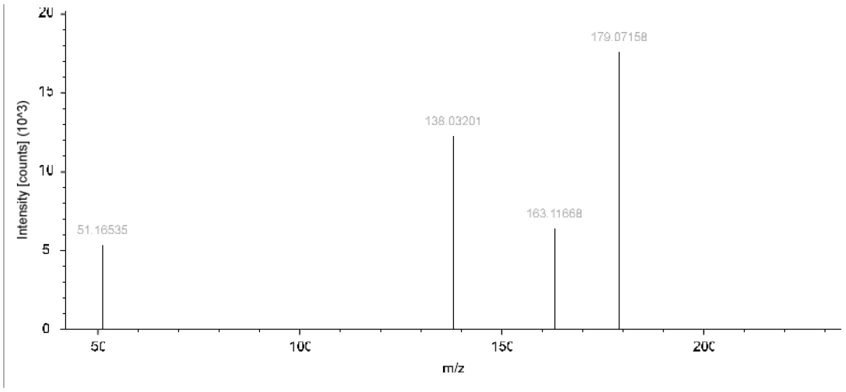

RT 8.2 MS2, FTMS(-), HCD, 223.0612 @ 30,50,70

M09: 1-(3‘,4‘,5‘-trihydroxyphenyl)-3-(2‘‘,4‘‘,6‘‘-trihydroxyphenyl)propan-2-ol

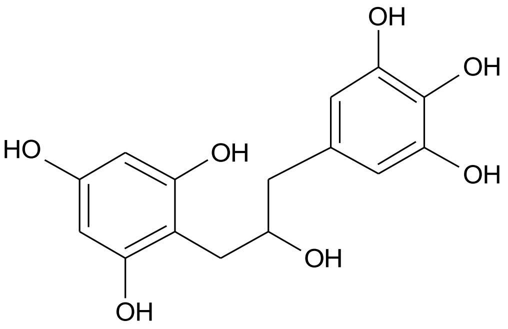

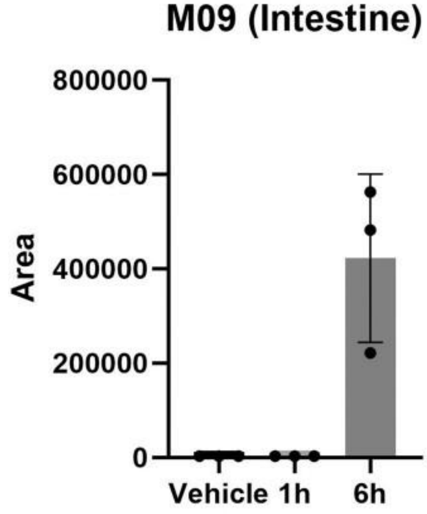

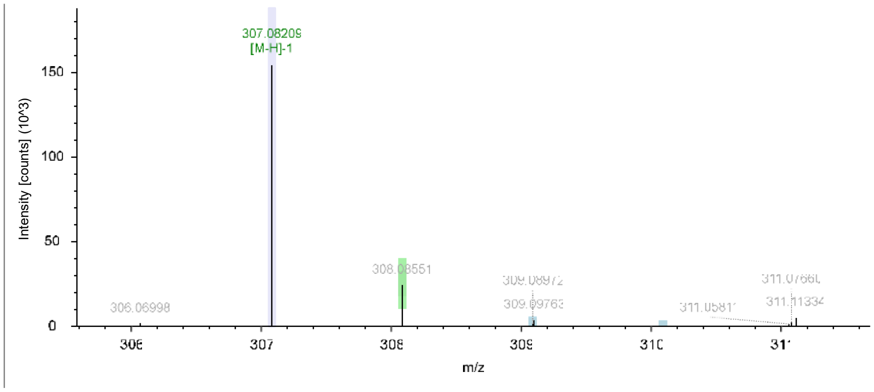

Negative full scan; RT 9.4 min

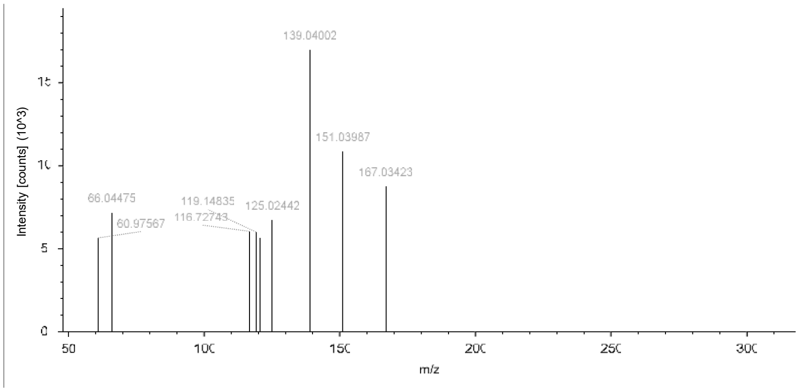

RT 9.4 MS2, FTMS(-), HCD, 307.0821 @ 30,50,70

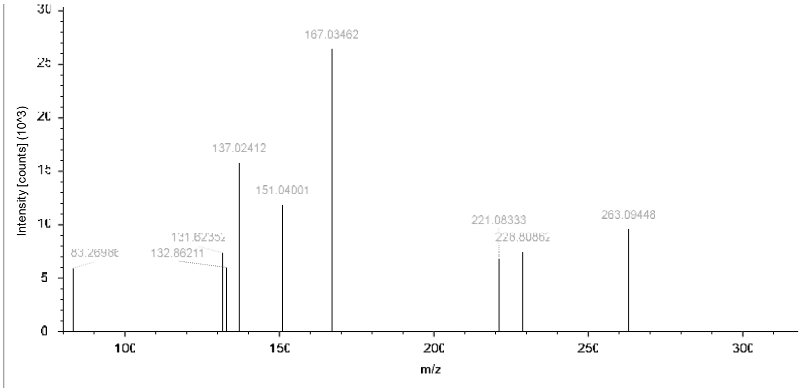

RT 9.4 MS2, FTMS(-), CID, 307.0821 @ 30

M10 Epigallocatechin

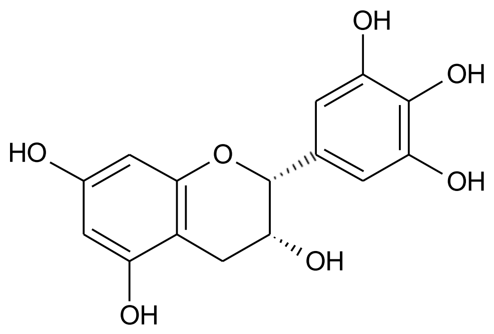

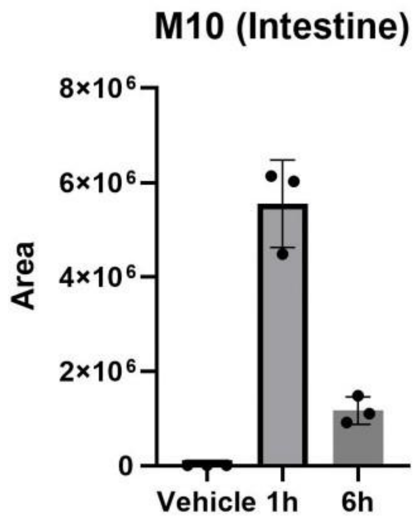

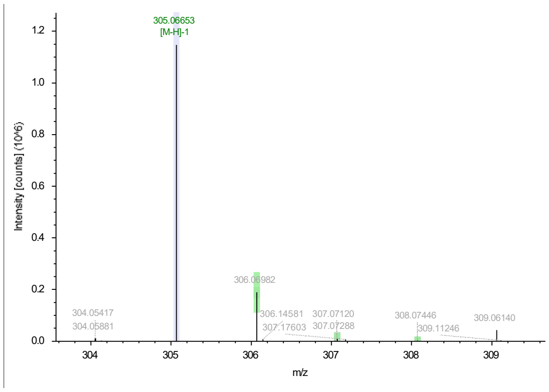

Negative full scan; RT 9.5 min

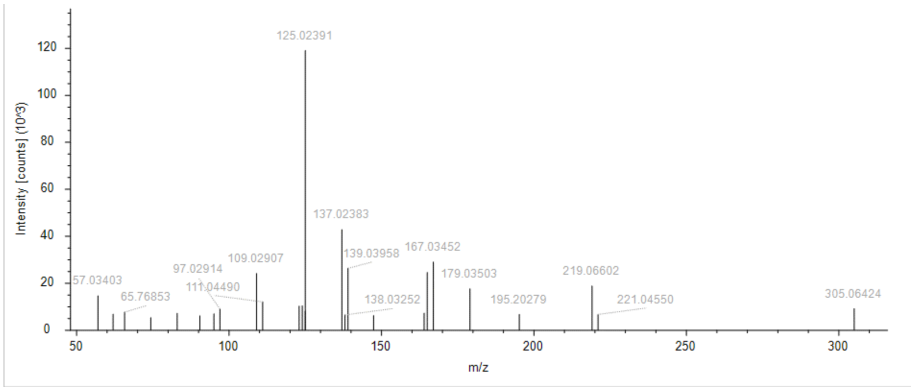

RT 9.5 MS2, FTMS(-), HCD, 305.0655 @ 30,50,70

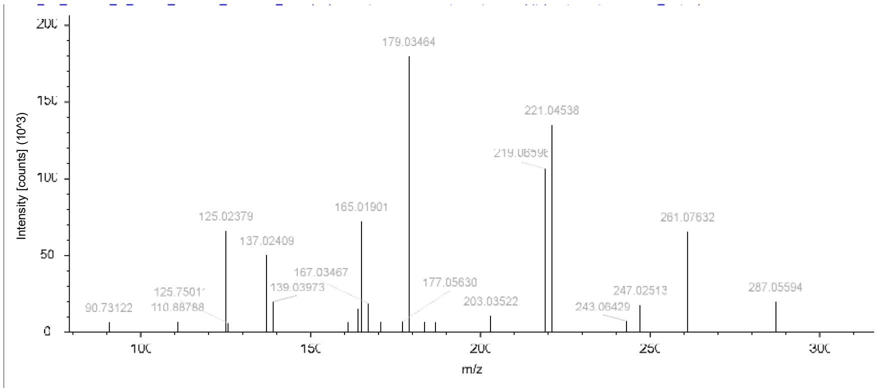

RT 9.5 MS2, FTMS(-), CID, 305.0655 @ 30

M11 Catechin:

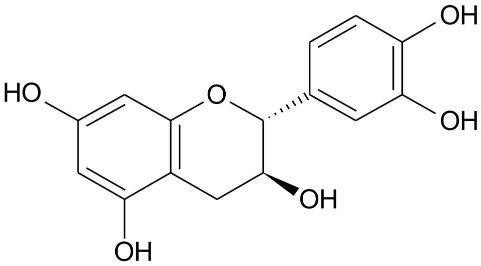

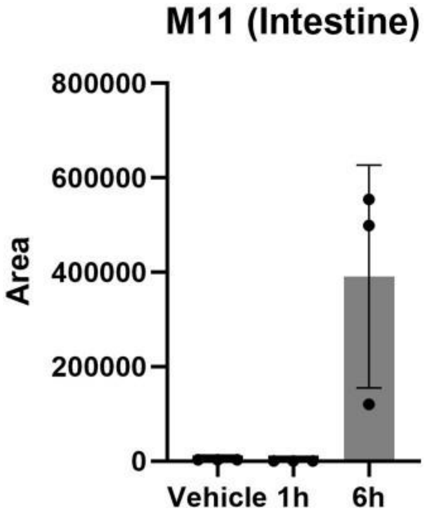

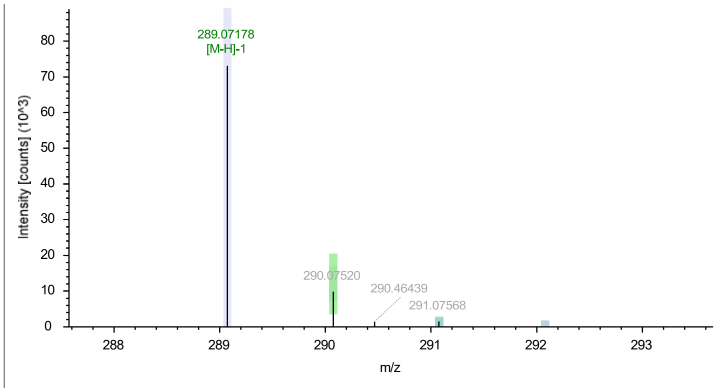

RT Negative full scan; RT 9.7 min

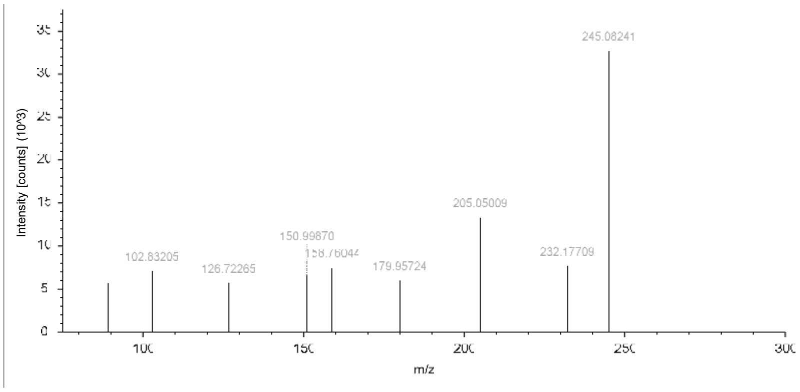

RT 9.7 MS2, FTMS(-), CID, 289.0717 @ 30

M12: 5-(3‘,5‘-Dihydroxyphenyl)-γ-Valerolactone

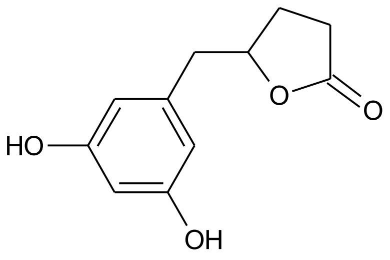

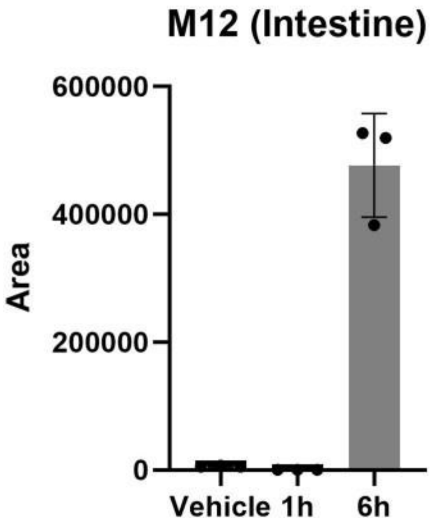

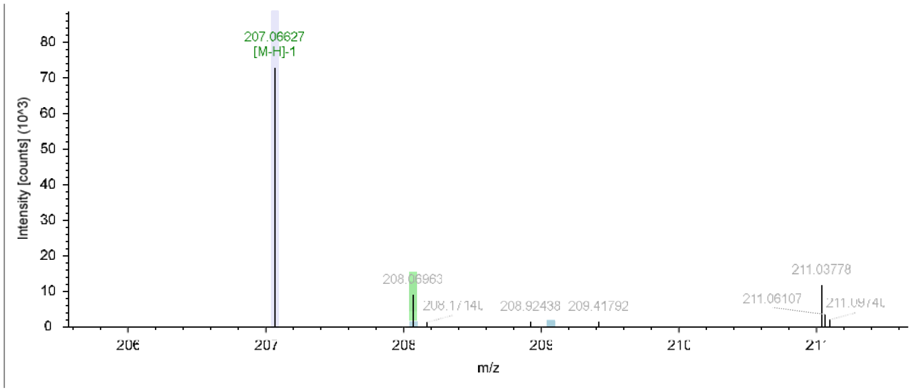

RT Negative full scan; RT 10.8 min

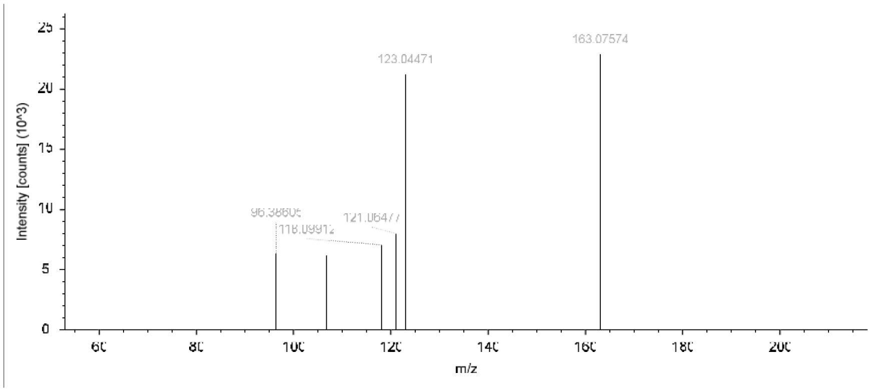

RT 10.8 MS2, FTMS(-), CID, 207.0663 @ 30

M13: 5-(3,4-Dihydroxyphenyl)-γ-Valerolactone

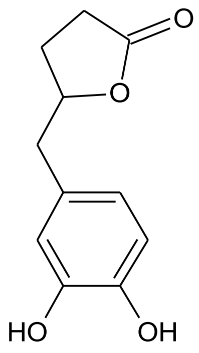

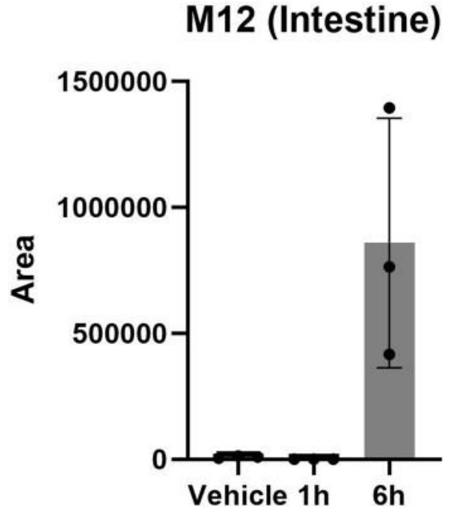

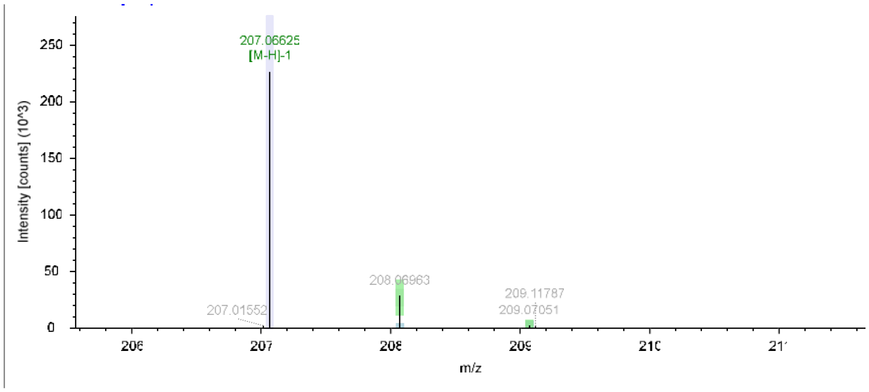

RT Negative full scan; RT 12.2 min

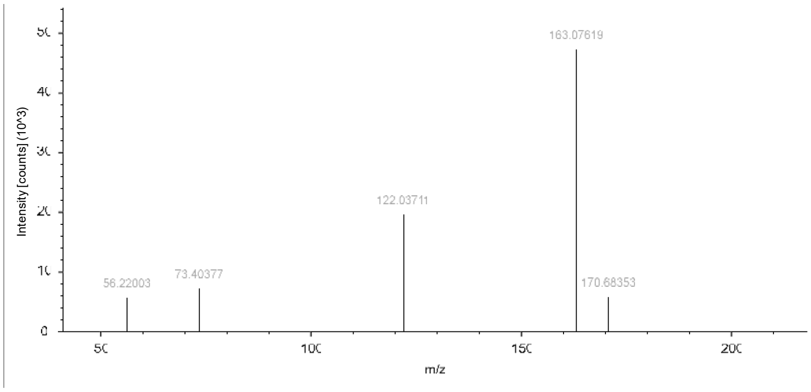

RT 12.2 MS2, FTMS(-), HCD, 207.0663 @ 30,50,70

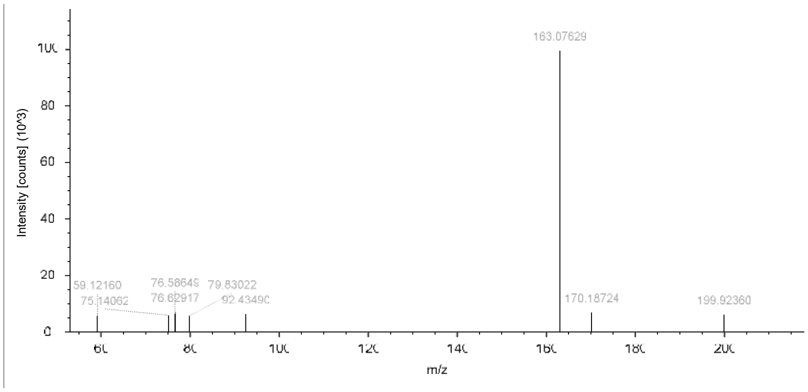

RT 12.2 MS2, FTMS(-), CID, 207.0663 @ 30

M14: 1-(3',5'-dihydroxyphenyl)-3-(2'',4'',6''-trihydroxyphenyl)propan-2-ol

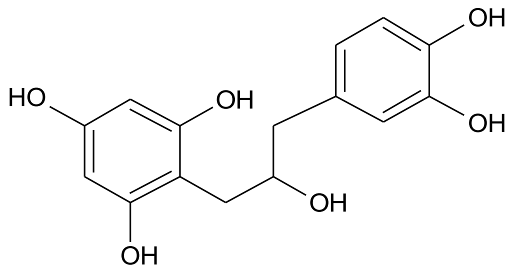

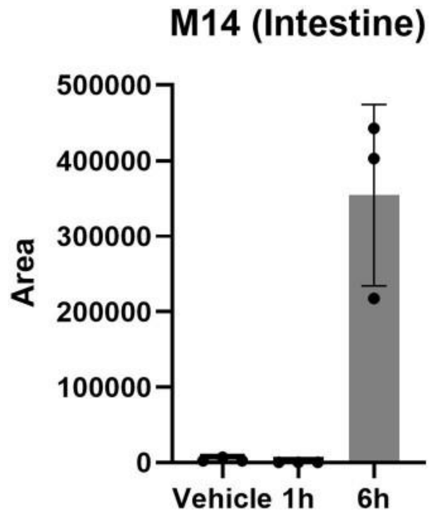

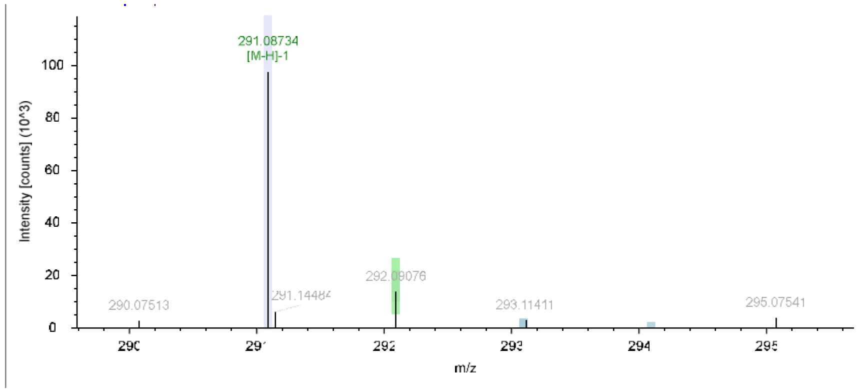

Negative full scan; RT 12.2 min

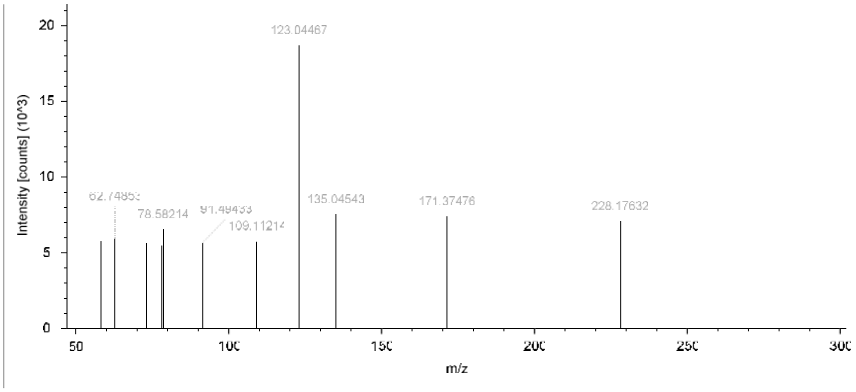

RT 12.2 MS2, FTMS(-), HCD, 291.0873 @ 30,50,70

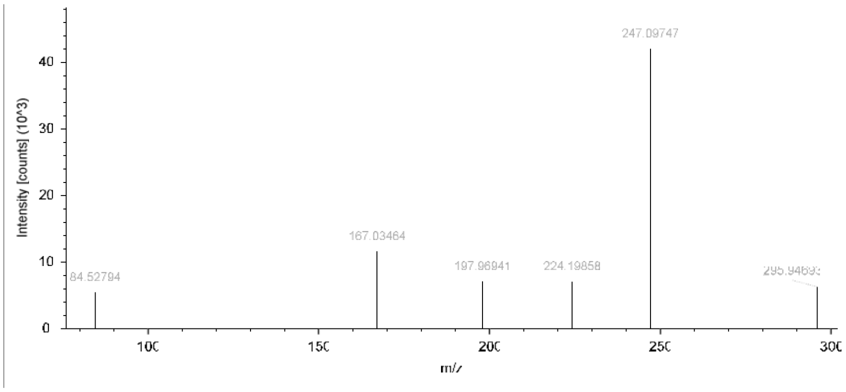

RT 12.2 MS2, FTMS(-), CID, 291.0873 @ 30

M15 Epigallocatechin gallate

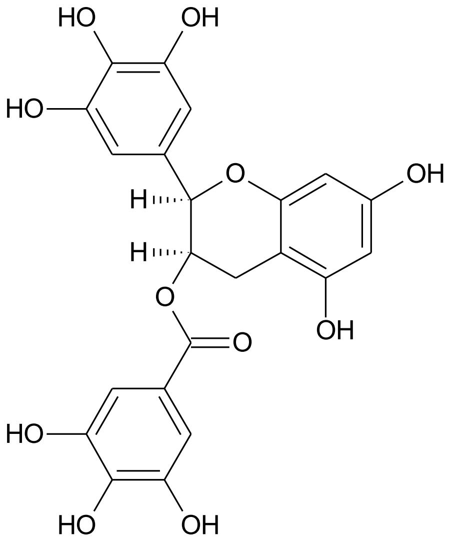

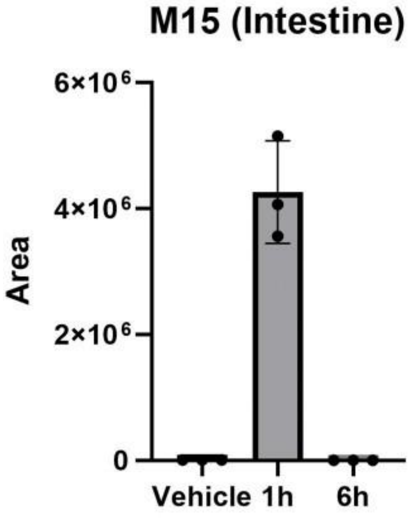

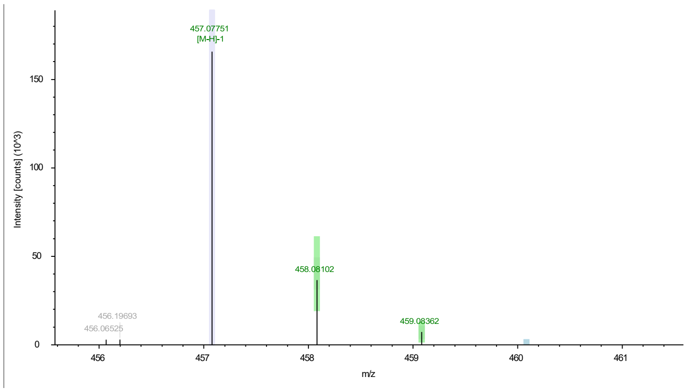

Negative full scan; RT 12.5 min

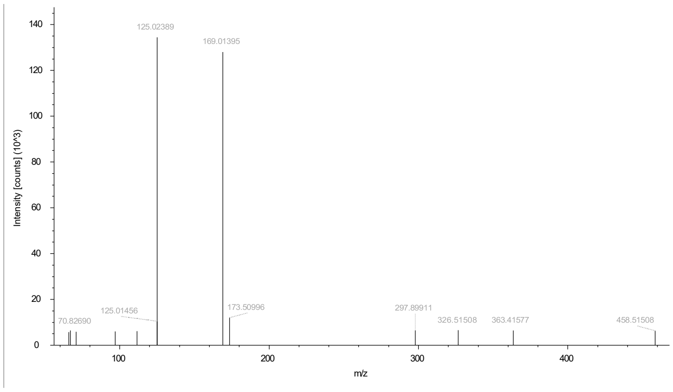

RT 12.5 MS2, FTMS(-), HCD, 457.0775 @ 30,50,70

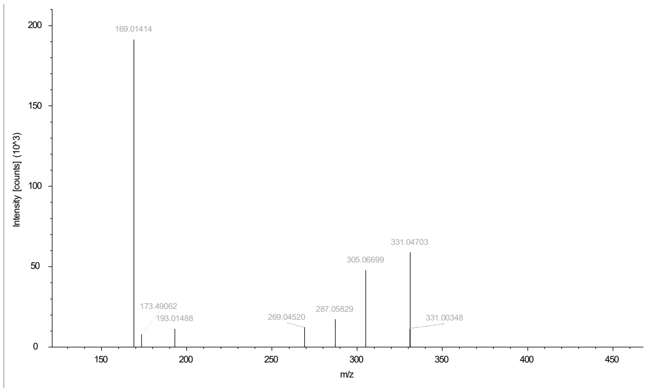

RT 12.5 MS2, FTMS(-), CID, 457.0775 @ 30

M16: No Identification

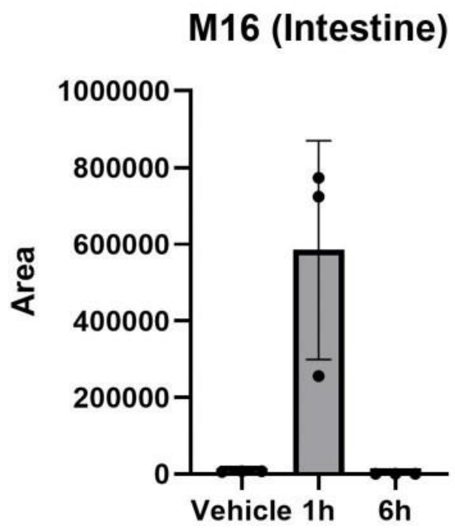

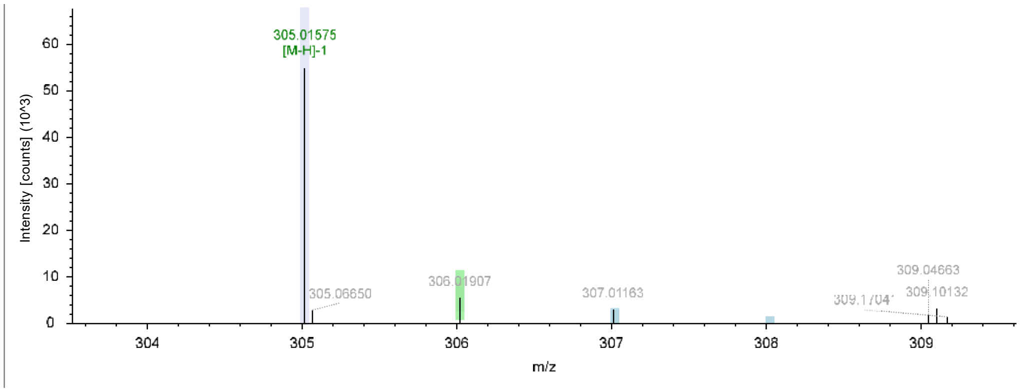

Negative full scan; RT 12.5 min

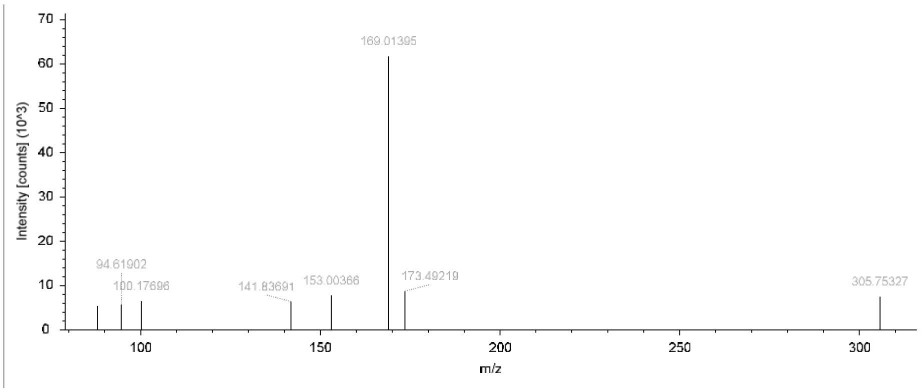

RT 12.5 MS2, FTMS(-), CID, 305.0157 @ 30

M17: (2Z)-5-(3,4-dihydroxy-5-methoxyphenyl)-4,5-dihydroxy-3-phenylpent-2-enoic acid

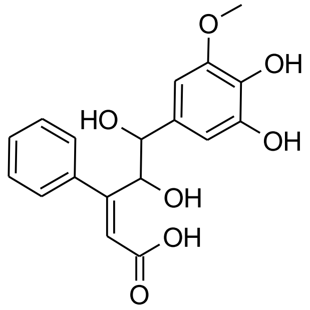

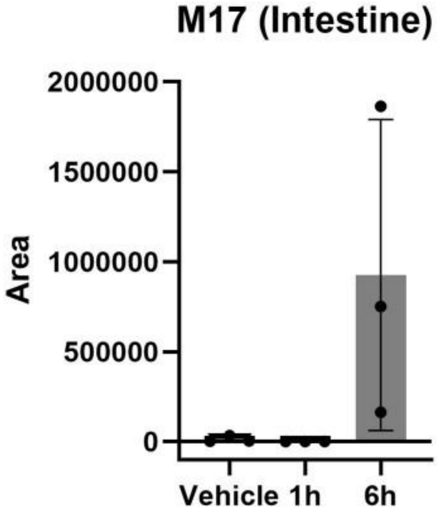

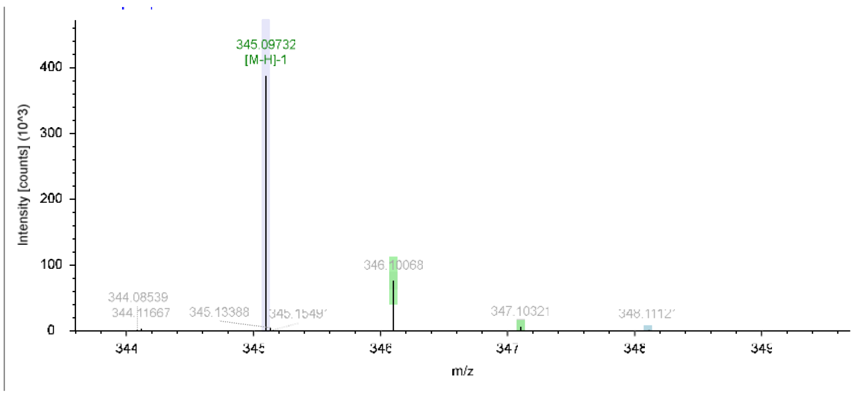

Negative full scan; RT 12.5 min

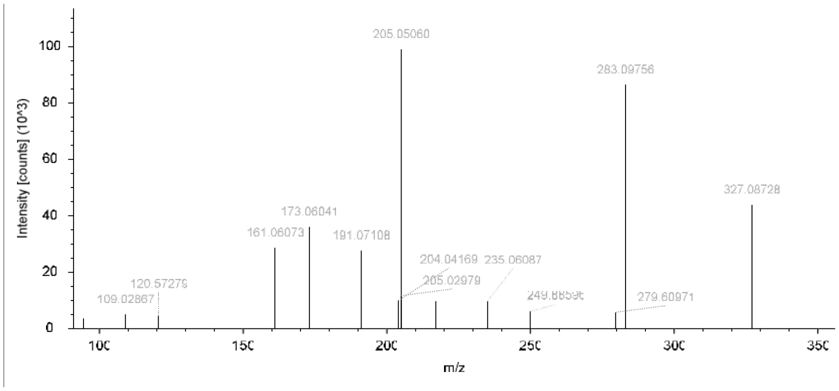

RT 12.5 MS2, FTMS(-), CID, 345.0973 @ 30

M18: Myricetin 3-O-glucuronide

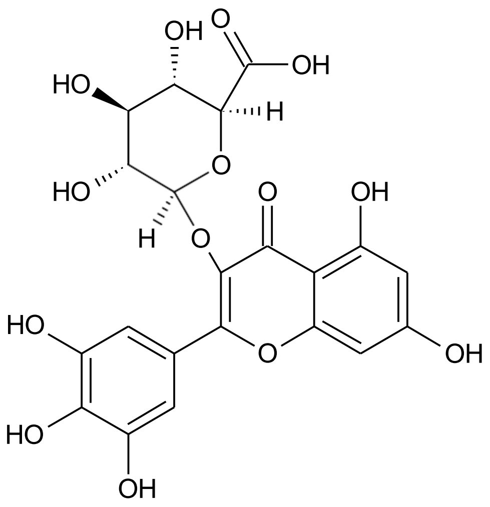

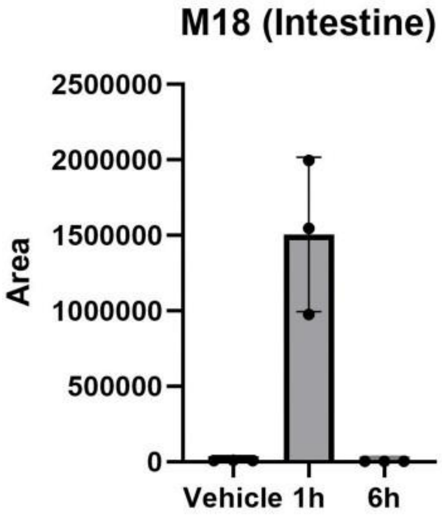

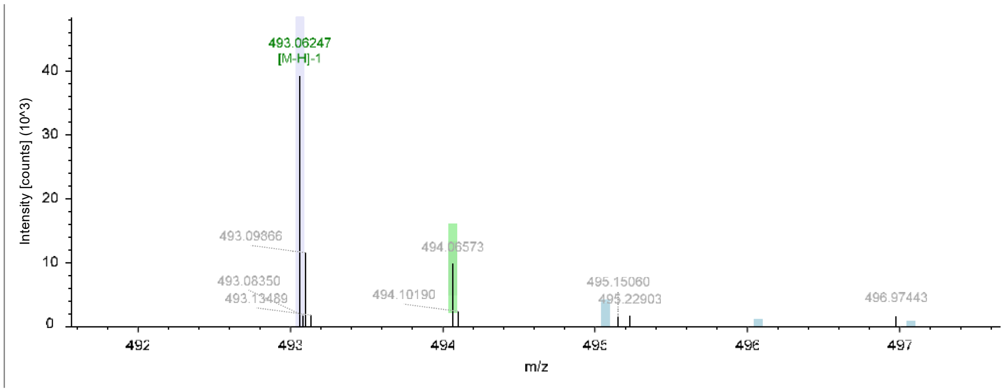

Negative full scan; RT 12.8 min

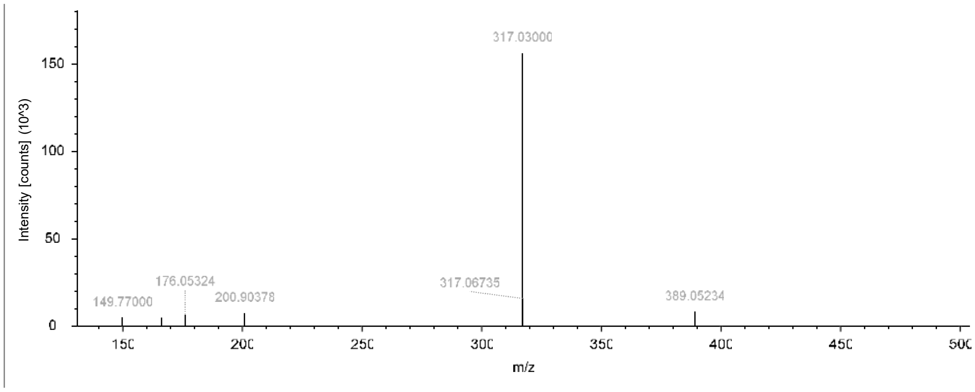

RT 12.8 MS2, FTMS(-), CID, 493.0625 @ 30

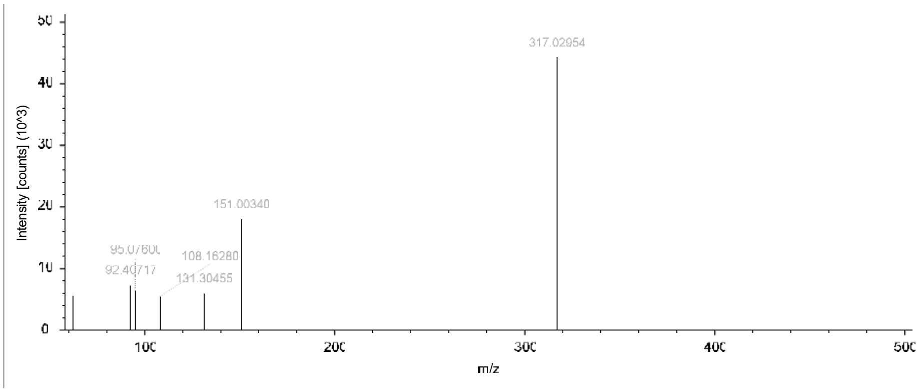

RT 12.8 MS2, FTMS(-), CID, 493.0625 @ 30,50,70

M19: 5'-O-methyl Myricetin 3-O-glucuronide

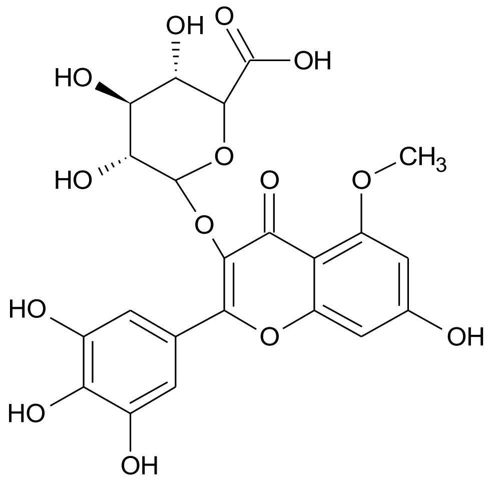

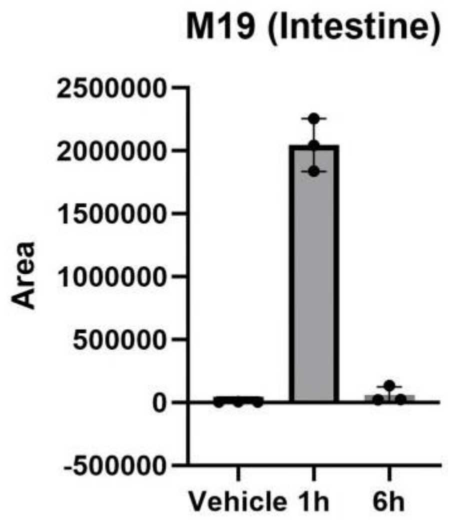

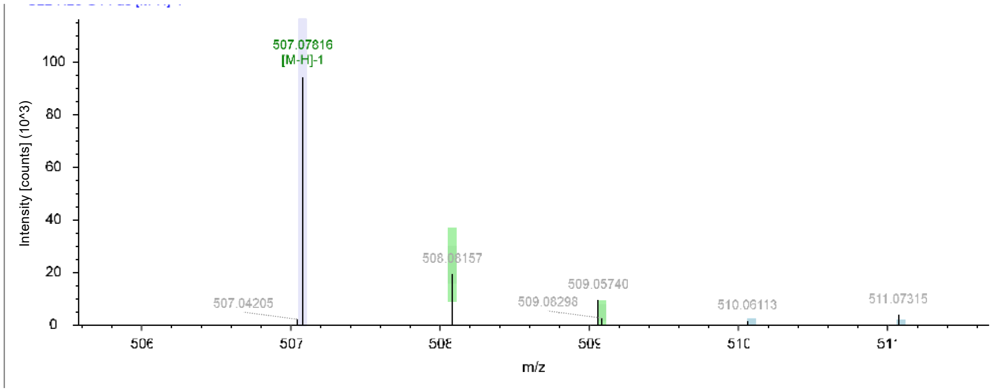

Negative full scan; RT 14.0 min

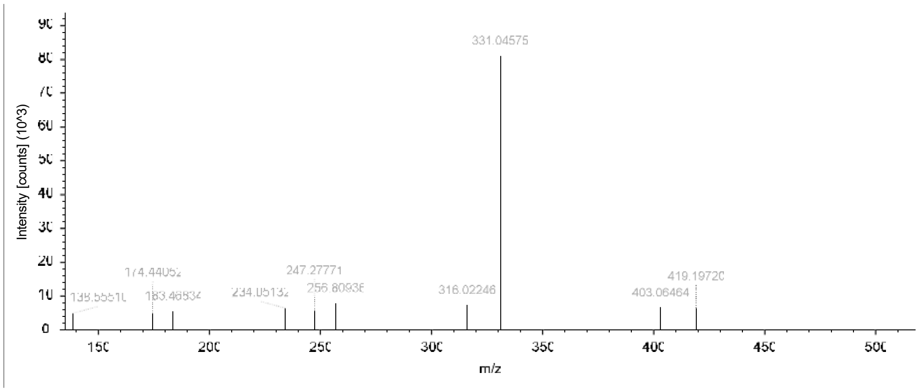

RT 14.0 MS2, FTMS(-), CID, 507.0782 @ 30

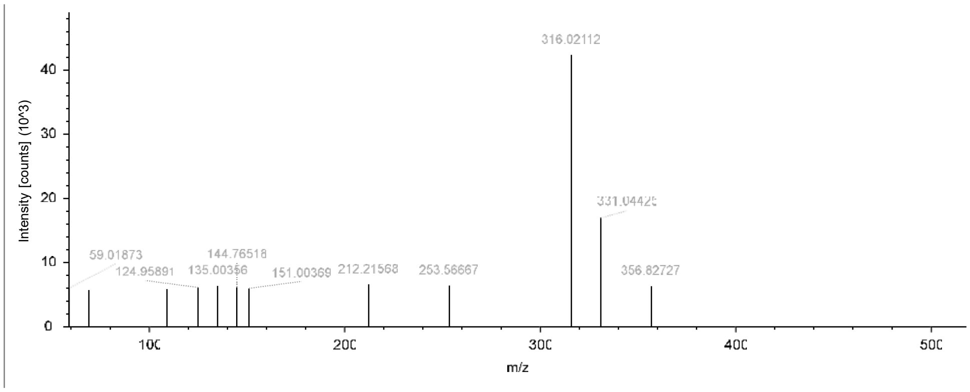

RT 14.0 MS2, FTMS(-), HCD, 507.0782 @ 30,50,70

M20 Myricetin

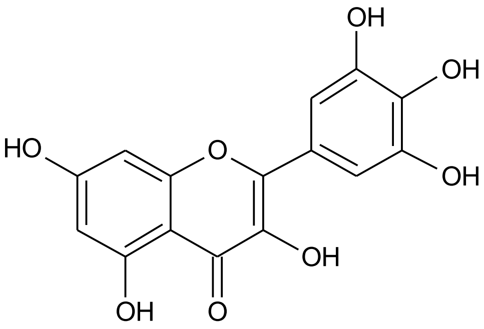

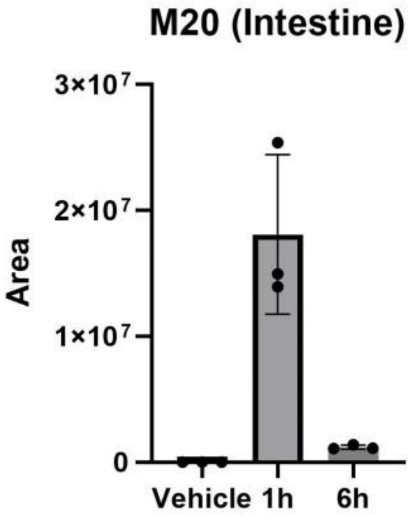

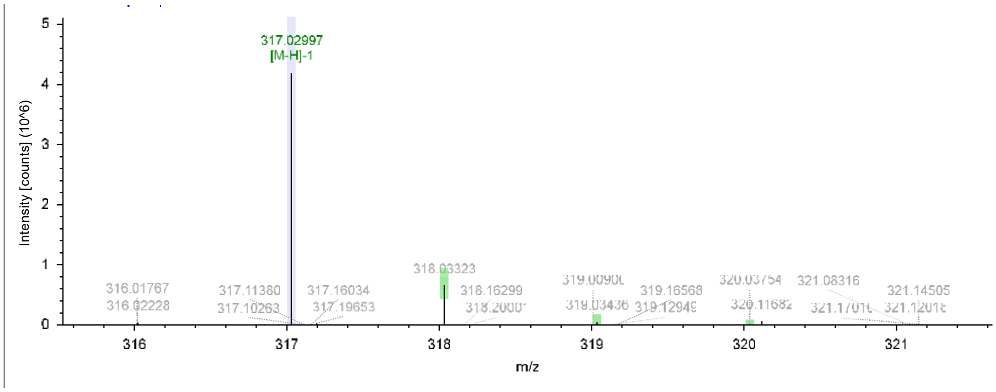

Negative full scan; RT 15.3 min

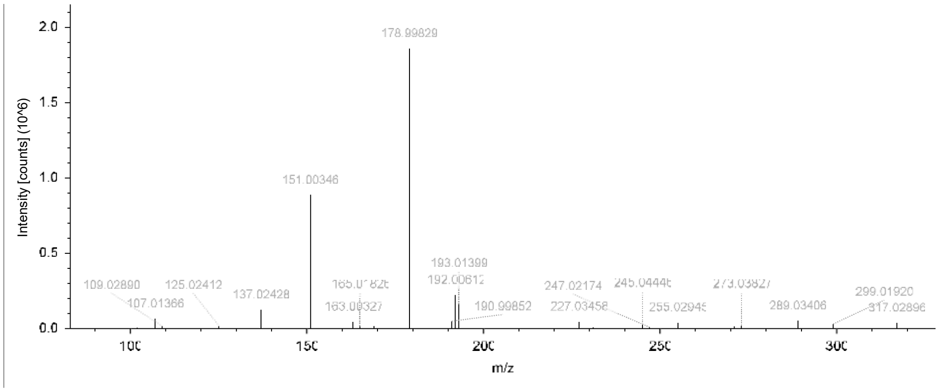

RT 15.3 MS2, FTMS(-), CID, 317.0300 @ 30,

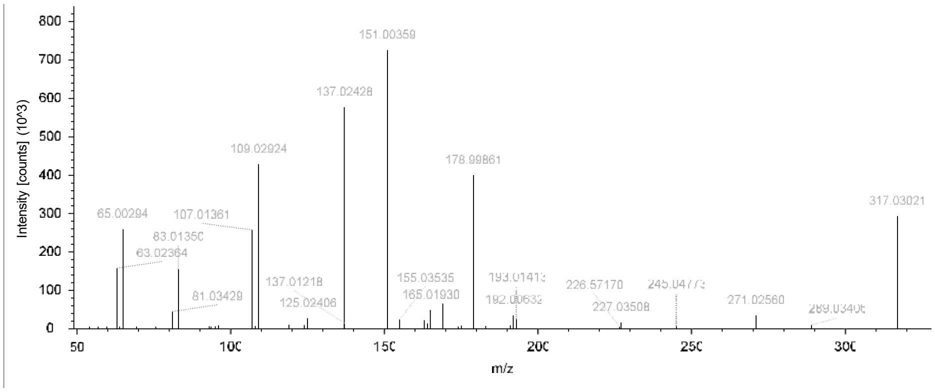

RT 15.3 MS2, FTMS(-), HCD, 317.0300 @ 30,50,70

M21: 5-O-Methylmyricetin

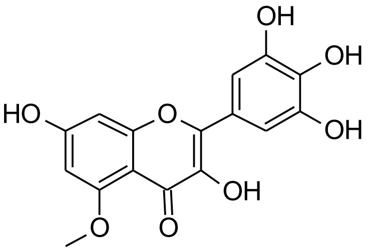

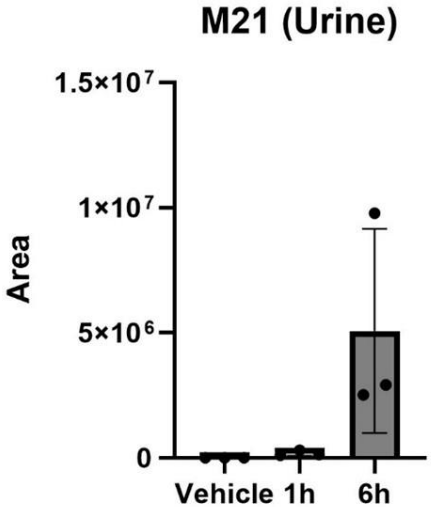

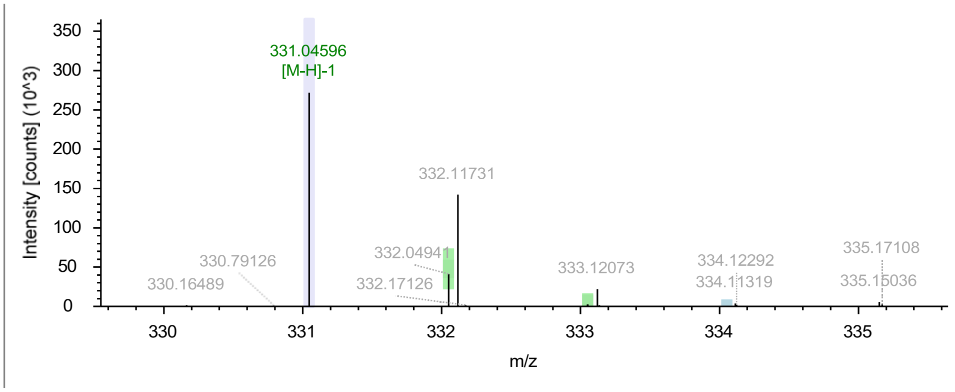

Negative full scan; RT 16.5 min

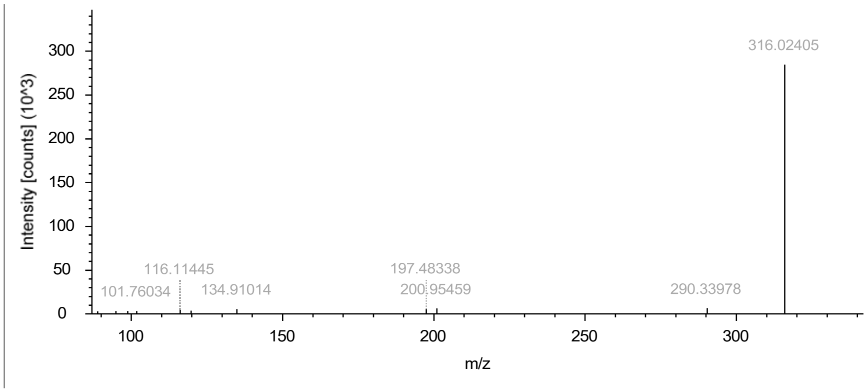

RT 16.5 MS2, FTMS(-), CID, 331.0460 @ 30

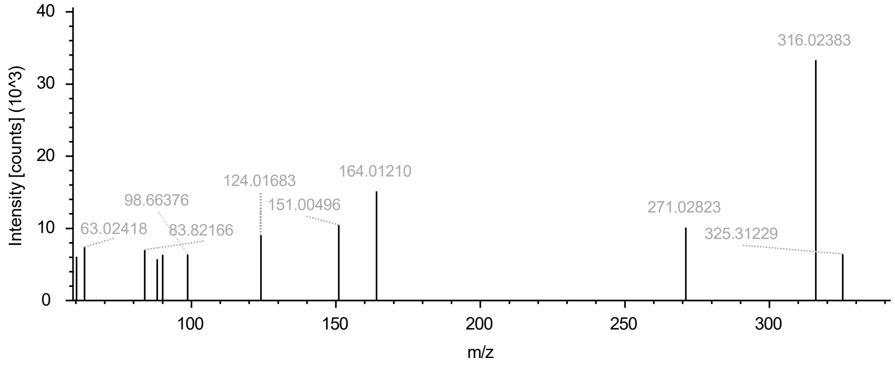

RT 16.5 MS2, FTMS(-), HCD, 331.0460 @ 30,50,70

M22: No Identification

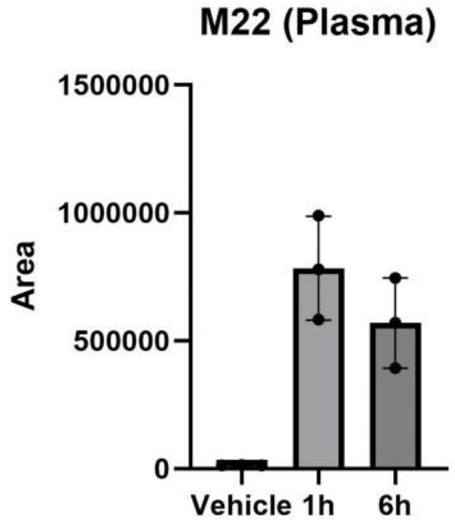

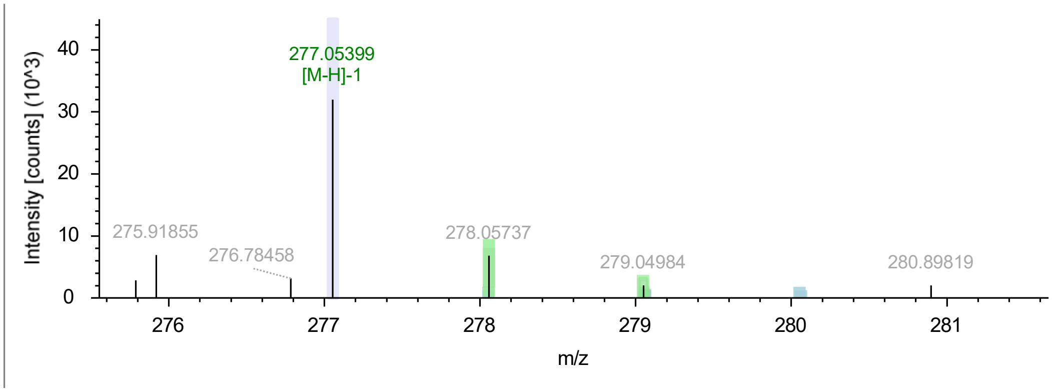

Negative full scan; RT 19.7 min

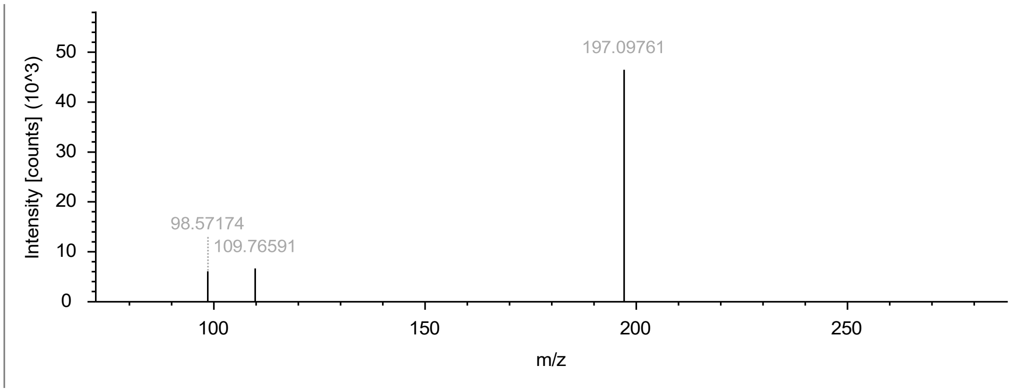

RT 19.7 MS2, FTMS(-), CID, 277.054 @ 30

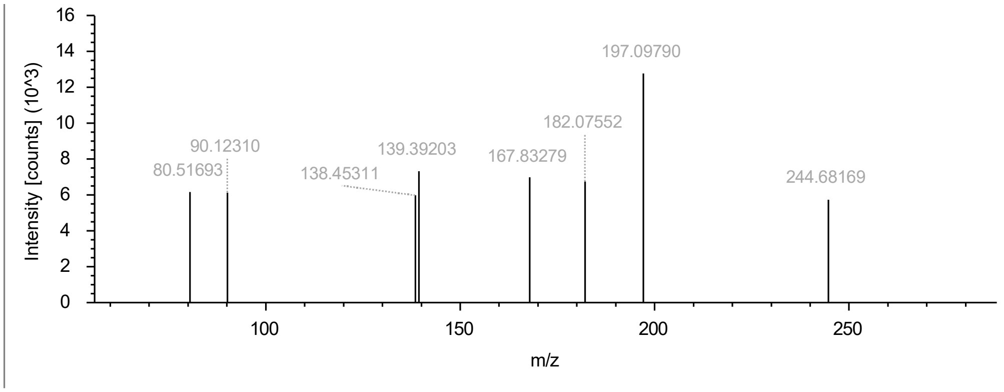

RT 19.7 MS2, FTMS(-), HCD, 277.054 @ 30,50,70

References

ELLMAN, G. L., COURTNEY, K. D., ANDRES, V., JR. & FEATHER-STONE, R. M. 1961. A new and rapid colorimetric determination of acetylcholinesterase activity. *Biochem Pharmacol,* 7**,** 88-95.

GERMER, S., RITTER, T. & WURGLICS, M. 2024. Substantial Differences in Proanthocyanidin Contents among Ginkgo biloba Leaf Extracts in Herbal Medicinal Products Obtained from the German Market. *Planta Med,* 90**,** 1040-1047.
